# Fc**γ**RIIB regulates autoantibody responses by limiting marginal zone B cell activation

**DOI:** 10.1172/JCI157250

**Published:** 2022-09-01

**Authors:** Ashley N. Barlev, Susan Malkiel, Izumi Kurata-Sato, Annemarie L. Dorjée, Jolien Suurmond, Betty Diamond

**Affiliations:** 1 Center of Autoimmune Musculoskeletal and Hematopoietic Diseases, The Feinstein Institutes for Medical Research, Northwell Health, Manhasset, New York, USA.; 2 Donald and Barbara Zucker School of Medicine at Hofstra/Northwell, Manhasset, New York, USA.; 3 Department of Rheumatology, Leiden University Medical Center, Leiden, Netherlands.

**Keywords:** Autoimmunity, Immunology, Adaptive immunity, Lupus

## Abstract

FcγRIIB is an inhibitory receptor expressed throughout B cell development. Diminished expression or function is associated with lupus in mice and humans, in particular through an effect on autoantibody production and plasma cell (PC) differentiation. Here, we analyzed the effect of B cell–intrinsic FcγRIIB expression on B cell activation and PC differentiation. Loss of FcγRIIB on B cells in *Fcgr2b*–conditional KO (*Fcgr2b*-cKO) mice led to a spontaneous increase in autoantibody titers. This increase was most striking for IgG3, suggestive of increased extrafollicular responses. Marginal zone (MZ) B cells had the highest expression of FcγRIIB in both mice and humans. This high expression of FcγRIIB was linked to increased MZ B cell activation, Erk phosphorylation, and calcium flux in the absence of FcγRIIB triggering. We observed a marked increase in IgG3^+^ PCs and B cells during extrafollicular PC responses in *Fcgr2b*-cKO mice. The increased IgG3 response following immunization of *Fcgr2b*-cKO mice was lost in MZ-deficient *Notch2* *Fcgr2b*–double KO mice. Importantly, patients with systemic lupus erythematosus (SLE) had a decrease in FcγRIIB expression that was strongest in MZ B cells. Thus, we present a model in which high FcγRIIB expression in MZ B cells prevented their hyperactivation and ensuing autoimmunity.

## Graphical abstract

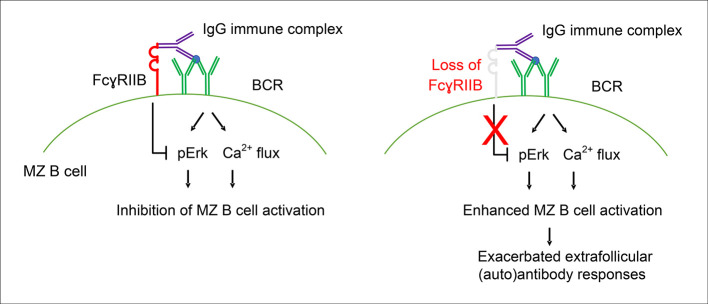

## Introduction

FcγRIIB is an inhibitory receptor expressed on many cell types, including B cells. On B cells, it is the only Fcγ receptor and is expressed throughout B cell development. When FcγRIIB is crosslinked to the B cell receptor (BCR), downstream BCR signaling and B cell activation are inhibited (1, 2). There are alleles of *FCGR2B* leading to loss of function or reduced expression that predisposes individuals to systemic lupus erythematosus (SLE) (3–6). The most common of these SNPs, the FcγRIIB-T232 variant, acts through loss of motility of FcγRIIB in the membrane, preventing proximity to the BCR (1, 6–8). Furthermore, reduced expression of FcγRIIB has been observed on CD27^+^ B cells from patients with SLE (9, 10).

In mice, global FcγRIIB deficiency was initially reported to cause a lupus-like disease, with the presence of autoantibodies and deposition of immune complexes in the kidney (11). However, there has been controversy surrounding the predisposition to lupus in FcγRIIB-deficient mice, as some studies suggest that other genes in concordance with *Fcgr2b* are required to influence the development of lupus (12, 13).

The effect of FcγRIIB on autoantibody production is B cell intrinsic, as overexpression of FcγRIIB on B cells from lupus-prone mice leads to improvement in the lupus phenotype (14, 15), and B cell–specific deletion of FcγRIIB leads to autoantibody production and a lupus phenotype (15, 16). FcγRIIB also plays an important role in aberrant responses to infection, such as to malaria (17).

Several studies have suggested a role for FcγRIIB in B cell tolerance, either through an effect on germinal center (GC) B cells, B-1 cells, or plasma cell (PC) survival (18–24). Mechanisms reported to contribute to the development of autoreactivity from the loss of FcγRIIB include activation of bystander autoreactive GC B cells, loss of follicular (FO) exclusion, and absence of PC apoptosis (25–27). Whereas most studies have focused on the role of FcγRIIB in the GC, recent developments have highlighted the role of extrafollicular B cell responses in SLE (28–30), necessitating an expanded analysis of the role of FcγRIIB in B cells.

We analyzed the role of FcγRIIB in B cell–specific (CD19-Cre) FcγRIIB-deficient mice, with a focus on extrafollicular responses. We show that loss of FcγRIIB in B cells led to aberrant marginal zone (MZ) activation and subsequent extrafollicular autoreactive PC responses.

## Results

### Increased spontaneous autoantibody IgG3 responses in Fcgr2b-cKO mice.

**Figure 1:**
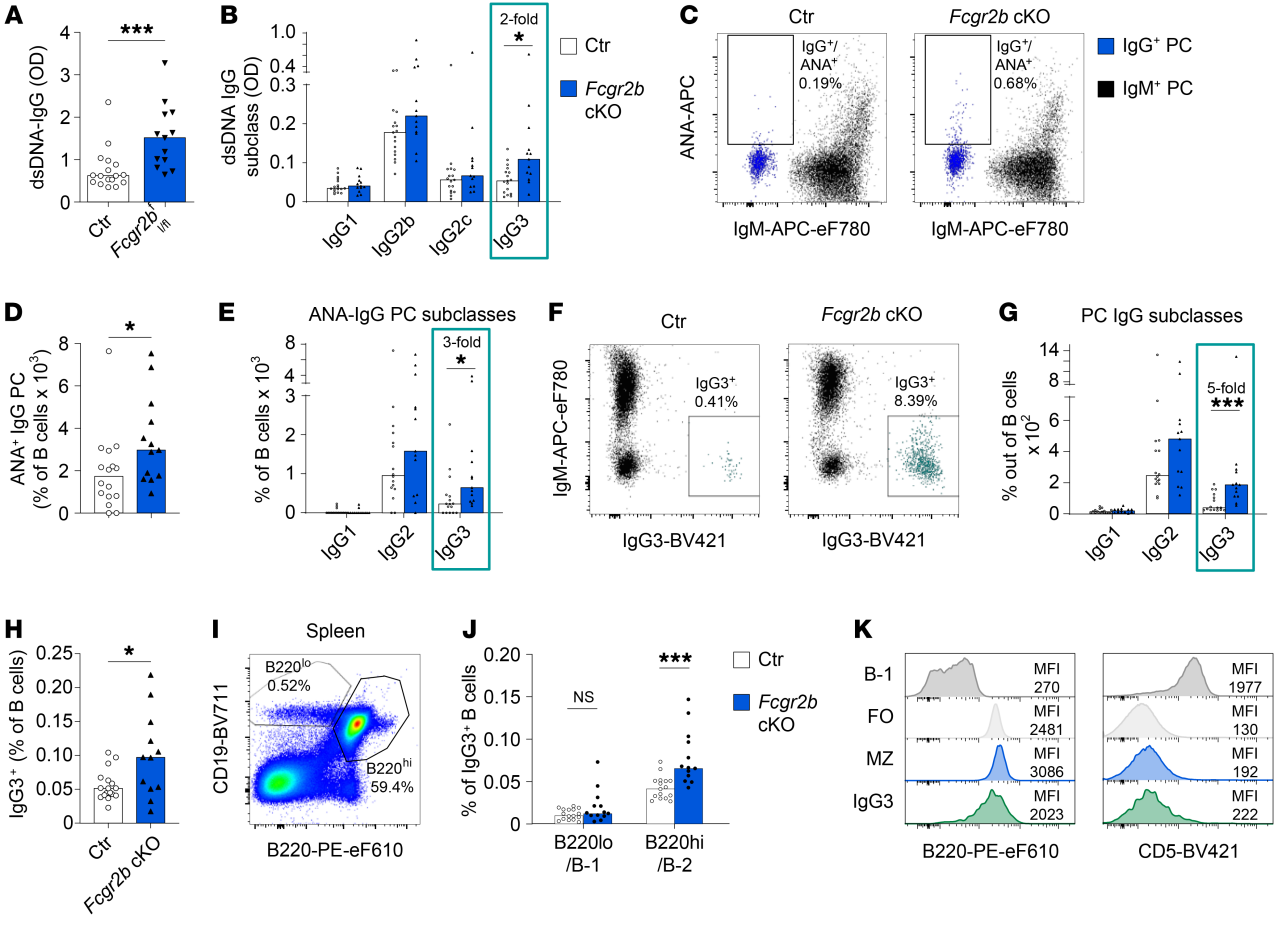
Increased spontaneous autoantibody IgG3 responses in *Fcgr2b*-cKO mice. Female control (Ctr) and *Fcgr2b*-cKO mice were bred and maintained until experiments at 10–12 months of age, at which point (auto)antibodies in serum and PCs in spleen were characterized. ANA reactivity of PCs was established using flow cytometry. (**A** and **B**) dsDNA ELISA for total IgG and IgG subclasses in serum from *Fcgr2b*-cKO mice. (**C**) Representative example of ANA staining in IgG and IgM PCs in spleen. (**D** and **E**) Frequency of ANA^+^ IgG^+^ PCs in spleen, total IgG (**D**), and by IgG subclass (**E**). (**F**) Representative example of IgM and IgG3 staining in total PCs. IgG3^+^ cells are indicated in green. (**G**) Frequency of IgG^+^ PCs in spleen separated by subclass. (**H**) Frequency of IgG3^+^ B cells in control and *Fcgr2b*-cKO mice. (**I**) Representative example of staining strategy for B-1 and B-2 cells in spleen. (**J**) Percentage of splenic IgG3^+^ B cells with a B-1 or B-2 phenotype, respectively, gated as in **I**. (**K**) Representative example of staining for B220 and CD5 in total IgG3^+^ B cells compared with B-1, FO, and MZ B cells in spleen. Data are shown as the median, with each symbol representing an individual mouse (**A**, **B**, **D**, **E**, **G**, **H**, and **J**) (*n =* 12–17 per group pooled from 2–3 independent experiments). *P < 0.05 and ***P < 0.001, by Mann-Whitney *U* test.

We first characterized the spontaneous autoantibody production in mice with a B cell–specific FcγRIIB conditional KO (*Fcgr2b*-cKO). As reported previously, we observed increased anti-dsDNA IgG, which was present in mice at around 4–5 months of age (Figure 1A and Supplemental Figure 1A; supplemental material available online with this article; https://doi.org/10.1172/JCI157250DS1). Interestingly, when analyzing the IgG subclasses, we observed a significant increase only in IgG3 anti-dsDNA (Figure 1B). Using a flow cytometric assay we developed to assess anti–nuclear antibody–positive (ANA^+^) PCs (31), we observed an increased frequency of ANA^+^IgG^+^ PCs in the spleens of *Fcgr2b*-cKO mice (Figure 1, C and D). In line with the serum data, the increase in ANA^+^IgG^+^ PCs was only significant in the IgG3 subclass (Figure 1E). The frequency of ANA^+^ PCs within other isotypes or in the BM was not increased (Supplemental Figure 1, B–F). The increase in ANA^+^IgG3^+^ PCs in the spleen suggested an extrafollicular B cell response.

Since we previously showed that increases in ANA^+^ IgG PCs in patients with SLE and lupus-prone mice occur through aberrant IgG PC differentiation rather than as a result of an antigen-specific tolerance defect (31), we also analyzed tolerance checkpoints for ANA^+^ B cells and PCs in *Fcgr2b*-cKO mice. B cell–intrinsic FcγRIIB deficiency did not affect the percentage of ANA^+^ mature naive B cells or more immature B cell subsets in the BM or spleen (Supplemental Figure 2, A–C and Supplemental Figure 3). In contrast, we detected a specific increase in the frequency of splenic IgG^+^ PCs and serum levels of total IgG (Supplemental Figure 1, G–I, M, and N). Again, the most prominent increase was observed in the IgG3 subclass, both in PCs and in serum titers (Figure 1, F and G, and Supplemental Figure 1, J–L and O–R).

Together, these results indicate that spontaneous (auto)antibody production occurred through enhanced differentiation or survival of IgG3 PCs.

*Fcgr2b*-cKO mice spontaneously displayed an increased frequency of resting IgG3^+^ B cells (Figure 1H and Supplemental Figure 4, A–E). IgG3 is usually derived from B-1 cells or MZ B cells, and increased numbers of peritoneal B-1 cells have been reported in complete *Fcgr2b^–/–^* mice (24). Most IgG3^+^ B cells in *Fcgr2b*-cKO mice had a B-2 (CD19^+^B220^hi^) phenotype and were CD5^–^ (Figure 1, I–K). In addition, the frequencies of B-1a or B-1b cells in the spleen and peritoneum were unaffected in *Fcgr2b*-cKO mice (Supplemental Figure 4, F–M), whereas MZ B cell frequencies were increased in *Fcgr2b*-cKO mice (Supplemental Figure 4, N and O).

### Increased extrafollicular PC responses.

**Figure 2:**
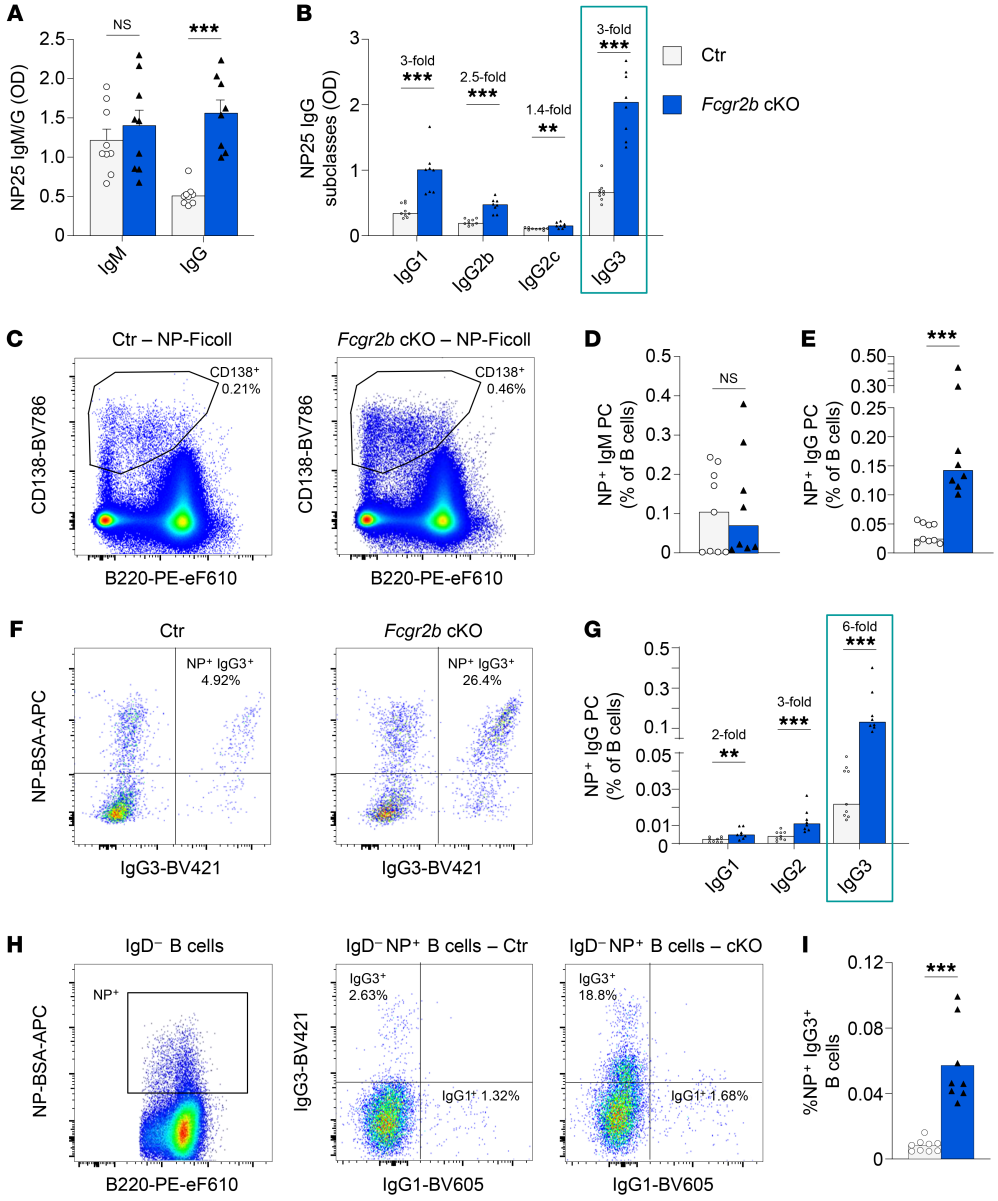
Increased extrafollicular responses following immunization. (**A**–**F**) Female control and *Fcgr2b*-cKO mice were immunized with NP-Ficoll. Serum and splenocytes were obtained 7 days later. (**A** and **B**) Levels of NP-specific antibodies, separated by isotype and subclass. (**C**) Representative example of PC staining following NP-Ficoll immunization (concatenated data on 4000 cells from 4 different mice per group). (**D** and **E**) Frequency of NP-specific PCs in spleen by isotype, as a percentage of B cells. (**F**) Representative examples of intracellular IgG3 and NP staining in splenic PCs. (**G**) Frequency of NP-specific PCs in spleen as a percentage of B cells, separated by IgG subclass. (**H**) Representative example of surface NP gating on IgD^–^ B cells (left) and IgG1 and IgG3 staining in IgD^–^NP^+^ B cells (middle and right; ~10,000 cells were concatenated from 4 mice per group). (**I**) Frequency of IgG3^+^NP^+^ B cells among total B cells. Data are shown as the median, with each symbol representing an individual mouse (*n =* 8–9 mice per group; data were pooled from 2–3 independent experiments). **P < 0.01 and ***P < 0.00, by Mann-Whitney *U* test.

IgG3 has been associated primarily with extrafollicular PC responses derived from MZ or B-1 cells (32–34). B-1 cells produce natural antibodies and can even produce these in the absence of antigen stimulation; and MZ B cells can be directly activated by antigens with repeated epitopes and TLRs and act as a first line of defense against blood-borne pathogens (24, 35, 36). Both of these cell types have been associated with extrafollicular humoral responses that are independent of cognate T cell help. Therefore, we analyzed the extrafollicular response to immunization with prototypical T-independent and T-dependent antigens, (4-hydroxy-3-nitrophenyl)acetyl–Ficoll (NP-Ficoll), and NP-CGG in alum, respectively. We observed a large increase in NP-IgG, but not NP-IgM, serum titers in *Fcgr2b*-cKO mice on day 7 following immunization with NP-Ficoll (Figure 2A). The anti-NP response of all IgG subclasses was significantly increased, with a marked increase in IgG3 (Figure 2B). In line with serum antibody levels, NP-specific IgG^+^ PCs in the spleen were increased, whereas no increase in IgM PCs was observed (Figure 2, C–E). The greatest increase in NP-specific PCs was present in the IgG3 subclass (Figure 2, F and G). Besides an increase in NP^+^IgG3^+^ PCs, there was an increased frequency of NP^+^IgG3^+^ B cells in the spleens of *Fcgr2b*-cKO mice (Figure 2, H and I).

Whereas total levels of NP-IgG and NP-IgM were not increased in *Fcgr2b*-cKO mice following a T-dependent response to NP-CGG (Supplemental Figure 5A), we observed an increase in NP-IgG3 serum levels and PCs (Supplemental Figure 5, B and C). Likewise, NP^+^IgG3^+^ B cells were increased (Supplemental Figure 5, D and E). In line with an extrafollicular origin of NP-specific IgG3 following NP-CGG immunization, low-affinity NP-25 IgG3 levels peaked early (around day 14), and high-affinity NP-2 IgG3 levels were detected only at low levels at all time points up to day 42 (Supplemental Figure 5, F and G).

Together, these data suggest that B cell–specific FcγRIIB deficiency led to greatly enhanced extrafollicular responses to immunization, with a particularly strong increase in IgG3 responses that may have been of MZ origin.

### Increased activation of MZ B cells in the absence of FcγRIIB.

**Figure 3:**
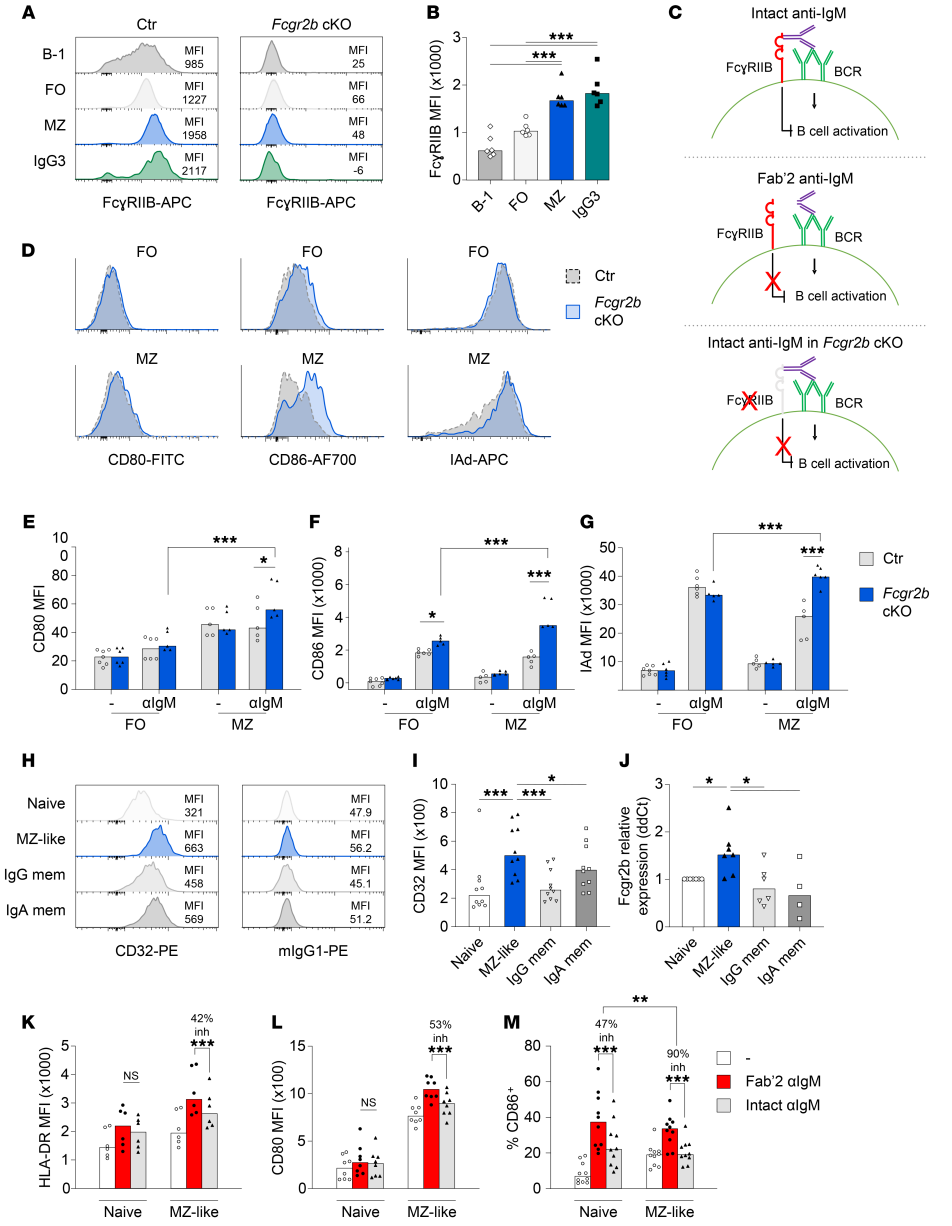
Phenotype and activation of MZ B cells in *Fcgr2b*-cKO mice and humans. (**A**) Representative example of staining for FcγRIIB in different B cell subsets and IgG3^+^ B cells in the spleen. Panel on the right shows staining in *Fcgr2b*-cKO cells. (**B**) FcγRIIB staining intensity in different B cell subsets from control mice. (**C**) Schematic of the experimental approach used to investigate the effect of FcγRIIB on B cell activation. Using intact anti-IgM, both the BCR and FcγRIIB are engaged (top); using Fab′2 anti-IgM (middle) or *Fcgr2b*-cKO cells (bottom), only the BCR is engaged. (**D**–**G**) Sorted FO and MZ B cells were stimulated with 3 μg/mL anti-IgM for 20 hours. Activation was measured by flow cytometry. Representative examples (**D**) and summary (**E**–**G**) of the expression of CD80, CD86, and IAd. (**H** and **I**) Representative examples and summary of expression of CD32 analyzed by flow cytometry using different human B cell subsets. (**J**) qPCR for *FCGR2B* in sorted human B cell subsets. Relative expression was normalized to polr2a, after which ΔΔCt was calculated compared with naive B cells. (**K**–**M**) Sorted human naive and MZ-like B cells were stimulated with 3 μg/mL intact anti-IgM or equimolar concentrations (2 μg/mL) of Fab′2 anti-IgM for 20 hours or were left untreated as controls. Upregulation of HLA-DR, CD80, and CD86 was measured by flow cytometry. The percentage of inhibition was calculated for each donor (the median percentage of inhibition is indicated). Data are shown as the median, with each symbol representing an individual mouse or human (*n =* 6 per group for **B**; *n =* 5–7 per group for **D**–**F**; *n =* 6–10 per group for **I**–**M**; data were pooled from 2–5 independent experiments; except for the data in **B**, which were from 1 experiment). *P < 0.05, **P < 0.01, and ***P < 0.001, by 1-way ANOVA with Bonferroni’s post hoc test (**B**, **I**, and **J**) or 2-way ANOVA with Bonferroni’s post hoc test (**E**–**G** and **K**–**M**). mem, memory B cells.

Since MZ or B-1 cells could be the origin of increased extrafollicular responses in *Fcgr2b-*cKO mice, we analyzed the expression of FcγRIIB in several B cell subsets in mice (Figure 3, A and B). We found that the expression of FcγRIIB was highest in MZ B cells and IgG3^+^ B cells compared with that in B-1 cells and FO B cells. Following immunization with NP-Ficoll and NP-chicken gamma globulin (NP-CGG), the we observed the highest expression of FcγRIIB in NP^+^IgG3^+^ B cells compared with expression in naive B cells or IgG1^+^NP^+^ B cells (Supplemental Figure 6, A–C). These results suggest that MZ B cells and IgG3^+^ B cells may be most susceptible to inhibition by FcγRIIB; stated otherwise: MZ B cells may be most activated in *Fcgr2b*-cKO mice.

Since we observed increased expression of FcγRIIB in MZ B cells, we asked whether there was a differential inhibitory effect of FcγRIIB on FO B cells and MZ B cells. Sorted B cell subsets were activated through BCR crosslinking in the presence (intact IgM crosslinking antibodies) or absence of FcγRIIB engagement (*Fcgr2b*-cKO cells or Fab′2 IgM crosslinking antibodies) (Figure 3C). We first determined optimal concentrations by Fab′2 IgM crosslinking antibodies for upregulation of CD80, CD86, and MHC class II (IAd) in FO B cells (Supplemental Figure 6, D–F) and observed increased upregulation of CD80 and CD86 in MZ B cells compared with FO B cells (Supplemental Figure 6, G–I). We then analyzed the effect of FcγRIIB engagement on BCR-mediated activation of FO and MZ B cells. We found that FcγRIIB deficiency increased the expression of CD80, CD86, and MHC class II in MZ B cells, whereas only CD86 was significantly upregulated in FO B cells in the absence of FcγRIIB (Figure 3, D–G). The absence of a strong effect on FO B cells was surprising, so we confirmed the observation by comparing equimolar concentrations of intact versus Fab′2 anti-IgM antibodies in control mice (Supplemental Figure 6, J–L).

To address these findings in humans, we analyzed the expression and inhibitory function of FcγRIIB in various B cell subsets from healthy donors. IgM^+^CD27^+^ B cells have been proposed as the human equivalent of MZ B cells in mice (MZ-like B cells) (37). We isolated these cells by FACS and confirmed their MZ-like phenotype by high *SOX7* and low *HOPX* expression, relative to conventional naive and IgG^+^ memory B cells (37) (Supplemental Figure 7, A and B). Next, we analyzed the expression of FcγRIIB in MZ-like B cells, naive B cells, and IgG and IgA memory B cells. As in mice, the human MZ-like compartment had the highest expression of FcγRIIB on both the protein and RNA level (Figure 3, H–J). MZ-like B cells were also more strongly activated than were naive B cells by BCR crosslinking using Fab′2 anti-IgM across a range of concentrations (Supplemental Figure 7, C–E). We next analyzed the effect of FcγRIIB-BCR crosslinking in MZ-like B cells compared with naive B cells and observed a stronger inhibitory effect of FcγRIIB engagement on MZ-like B cells than on naive B cells (Figure 3, K–M, and Supplemental Figure 7I). For example, although CD86 upregulation by BCR engagement was similar between naive B cells and MZ B cells (Supplemental Figure 7H), the inhibitory effect of FcγRIIB on MZ-like B cells returned CD86 expression close to baseline levels (Figure 3M). Upregulation of HLA-DR and CD80 upon BCR crosslinking was also stronger in MZ-like B cells (Supplemental Figure 7, F and G), and FcγRIIB-mediated inhibition was most pronounced in this subset (Figure 3, K and L).

MZ-like B cells have been reported to be more prone to activation; we now show that they also exerted stronger inhibition of activation through FcγRIIB, an effect that was observed in both mice and humans.

### FcγRIIB inhibits MZ B cell activation through inhibition of Erk phosphorylation and calcium flux.

**Figure 4:**
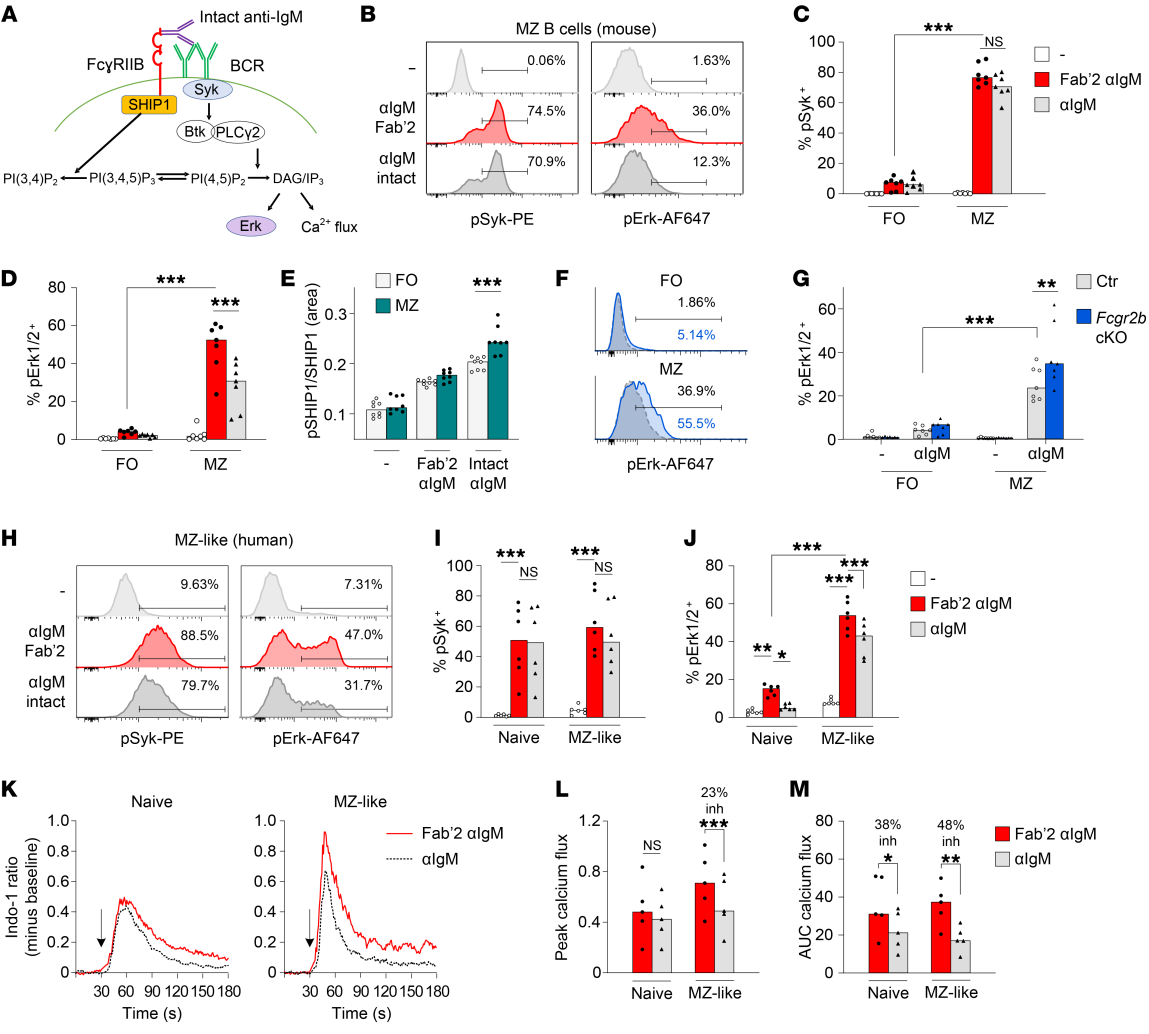
The effect of FcγRIIB on B cell signaling in MZ B cells. (**A**) Simplified schematic diagram of key signaling molecules downstream of the BCR and FcγRIIB. (**B**–**D**) Representative examples and summary of FO and MZ B cells from control mice; cells were stimulated with 3 μg/mL intact anti-IgM (αIgM) or equimolar concentrations (2 μg/mL) of Fab′2 anti-IgM for 10 minutes, followed by Phosphoflow analysis of the phosphorylation of signaling molecules. (**E**) SHIP1 phosphorylation was analyzed by capillary Western blotting. (**F** and **G**) Representative examples and summary of p-Erk expression in FO and MZ B cells from control and *Fcgr2b*-cKO mice; cells were stimulated with intact anti-IgM as described in **B**–**D**. (**H**–**J**) PBMCs from healthy donors were stimulated with 15 μg/mL intact anti-IgM or equimolar concentrations (10 μg/mL) of Fab′2 anti-IgM for 60 minutes, after which the phosphorylation of signaling molecules was analyzed by Phosphoflow. (**H**) Representative example of Syk and Erk phosphorylation in MZ-like B cells. (**I** and **J**) Comparison of intact versus Fab′2 anti-IgM in naive and MZ-like B cells. The median percentage of inhibition calculated per donor is indicated. (**K**–**M**) Calcium flux following 75 μg/mL intact anti-IgM or equimolar concentrations (50 μg/mL) of Fab′2 anti-IgM. Peak calcium flux and the AUC were calculated using FlowJo. (**K**) Representative examples of calcium flux in naive and MZ-like B cells. (**L** and **M**) Comparison of the peak and AUC of calcium flux following intact versus Fab′2 anti-IgM in naive and MZ-like B cells. inh, inhibition. Data are shown as the median, with each symbol representing an individual mouse (*n =* 4–7 mice per group for **B**–**D**; *n =* 8 mice per group for **G**; *n =* 6 mice per group for **H**–**J**; *n =* 5 mice per group for **L** and **M**; data for each were pooled from 2–3 independent experiments). *P < 0.05, **P < 0.01, and ***P < 0.001, by 2-way ANOVA with Bonferroni’s post hoc test.

We next analyzed the phosphorylation of signaling molecules downstream of the BCR and FcγRIIB (Figure 4A). We chose to study Syk, SHIP1, and Erk1/2 signaling. Syk is an early B cell signaling molecule that is not inhibited by FcyRIIB or SHIP1, the phosphatase activated by FcyRIIB engagement. Erk1/2 is downstream of FcyRIIB/SHIP1 signaling and is important for B cell activation and PC differentiation (38). Consistent with the increased upregulation of activation markers, we found increased phosphorylation of Syk and Erk1/2 in murine MZ B cells compared with FO B cells stimulated with Fab′2 anti-IgM (Figure 4, C and D). There was strong inhibition of Erk1/2 phosphorylation in MZ B cells in the presence of *Fcgr2b* engagement with intact anti-IgM antibodies, but no inhibition of Syk phosphorylation (Figure 4, B–D). A significant decrease in Erk1/2 phosphorylation was only observed in MZ B cells and not in FO B cells (Figure 4D). Furthermore, MZ B cells exhibited increased SHIP1 phosphorylation following combined BCR-FcγRIIB crosslinking compared with FO B cells (Figure 4E). Similar results for Erk1/2 phosphorylation were obtained when we studied the absence of FcγRIIb engagement in *Fcgr2b*-cKO mice compared with control mice (Figure 4, F and G). Again, this difference was only observed in MZ B cells.

In human MZ-like B cells, we likewise observed increased phosphorylation of Erk1/2 following BCR triggering in MZ-like cells compared with naive B cells (Supplemental Figure 8, A and B). FcγRIIB engagement strongly inhibited p-Erk in MZ-like B cells. Decreased Erk phosphorylation was also observed in naive B cells (Figure 4, H–J). MZ-like B cells compared with naive B cells had increased calcium flux upon BCR triggering (Supplemental Figure 9), and the calcium flux, particularly the peak flux, was more strongly inhibited by FcγRIIB in MZ-like B cells than in naive B cells (Figure 4, K–M).

These results point to a strong inhibitory effect of FcγRIIB on the activation of MZ B cells through decreased phosphorylation of Erk1/2 and decreased calcium flux.

### IgG3 responses in Fcgr2b-cKO mice are derived from MZ B cells.

**Figure 5:**
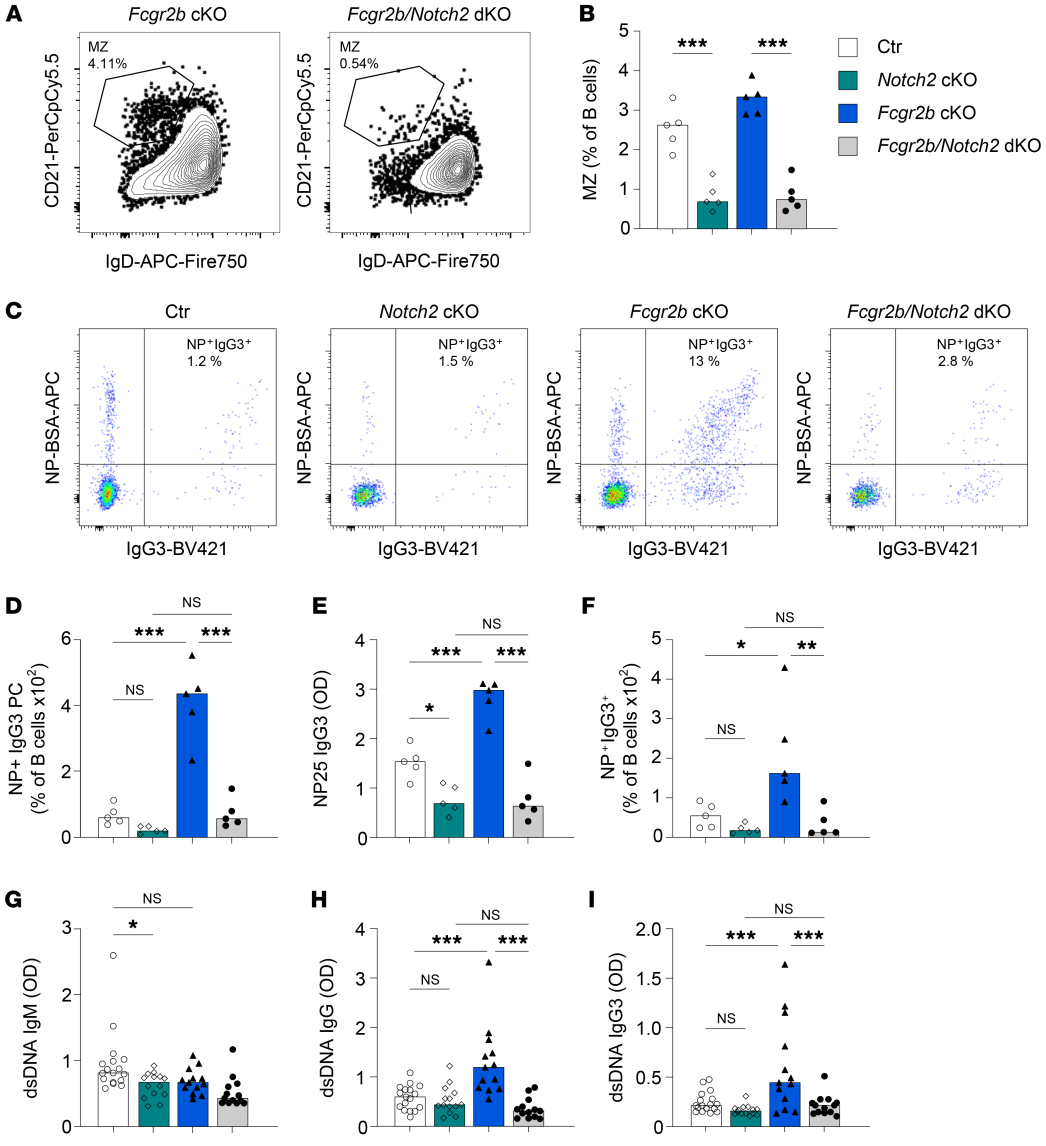
Combined FcγRIIB and MZ deficiency reverses the enhanced response to antigen challenge and the increase in autoantibody production. (**A**–**F**) Female control, *Notch2*-cKO, *Fcgr2b*-cKO, and *Notch2* *Fcgr2b*–dKO mice were immunized with NP-Ficoll. Serum and splenocytes were obtained 7 days later. (**A** and **B**) Representative examples of MZ B cell frequencies in *Fcgr2b*-cKO and *Notch2* *Fcgr2b*–dKO mice. (**C**) Representative examples of intracellular IgG3 and NP staining in splenic PCs. (**D**) Frequency of NP-specific IgG3^+^ PCs in spleen, as a percentage of B cells. (**E**) Levels of NP-specific IgG3 in serum. (**F**) Frequency of NP-specific IgG3^+^ B cells in spleen. (**G**–**I**) Female control, *Notch2*-cKO, *Fcgr2b*-cKO, and *Notch2* *Fcgr2b*–dKO mice were bred and maintained until 6–7 months of age, after which dsDNA antibodies in serum were characterized. dsDNA ELISAs for total IgM, IgG, and IgG subclasses were performed. Data are shown as the median, with each symbol representing an individual mouse (*n =* 5 mice per group for **A**–**F**; *n =* 13–17 mice per group for **G**–**I**). *P < 0.05, **P < 0.01, and ***P < 0.001, by 2-way ANOVA with Bonferroni’s post hoc test.

Because our results suggest that MZ B cells are a likely source for the increased IgG3 response in *Fcgr2b*-cKO mice, we generated double-KO (dKO) mice that lacked Notch2 and FcγRIIB in B cells, resulting in large reductions in MZ B cell numbers (39) and B cell–specific FcγRIIB deficiency. These mice exhibited deficiency of MZ B cells (~80% reduction), whereas FO B cells and B-1 cell numbers were maintained (Figure 5, A and B, and Supplemental Figure 9, A–C). Similar to what has been previously described (32), *Notch2*-cKO mice had decreased NP-specific IgM and IgG serum levels and PC numbers, but there was no effect of FcγRIIB on the IgM response in either the presence or absence of Notch2 (Supplemental Figure 9, D and F). More important, the increased IgG response to NP-Ficoll that we observed in *Fcgr2b*-cKO was completely reversed in the dKO mice lacking both Notch2 and FcγRIIB in B cells (Supplemental Figure 9, E and G). Furthermore, the increase in NP-IgG3 serum titers and NP^+^IgG3^+^ B cells and PCs generated by FcγRIIB deficiency was absent in dKO mice (Figure 5, C–F). IgG3^+^ B-2 B cells, but not IgG3^+^ B-1 B cells, were significantly reduced in MZ-deficient dKO mice (Supplemental Figure 9, H and I). Finally, we analyzed spontaneous autoantibody production triggered by deficiency of FcγRIIB in MZ-deficient mice. The increased IgG and IgG3 anti-dsDNA observed in FcγRIIB-cKO mice was abolished in dKO mice lacking both FcγRIIB and MZ B cells (Figure 5, G–I). Together, these results show that MZ B cells were responsible for enhanced extrafollicular (auto)antibody responses in *Fcgr2b*-cKO mice.

### Diminished expression of FcγRIIB in MZ-like B cells from patients with SLE.

Previous studies have shown diminished expression of FcγRIIB on CD27^+^ B cells from patients with SLE (9, 10). However, these cell populations were not studied in further detail, and CD27^+^ cells comprise both switched memory B cells as well as MZ-like B cells. We therefore wanted to assess the expression of FcγRIIB in MZ-like B cells from patients with SLE. We performed high-dimensional spectral flow cytometry, which allowed a detailed identification of more than 10 B cell subsets, in combination with staining for CD32B/C, on PBMCs from patients with SLE (*n =* 15) and healthy donors (*n =* 10). The patients’ characteristics are shown in Supplemental Table 2.

**Figure 6:**
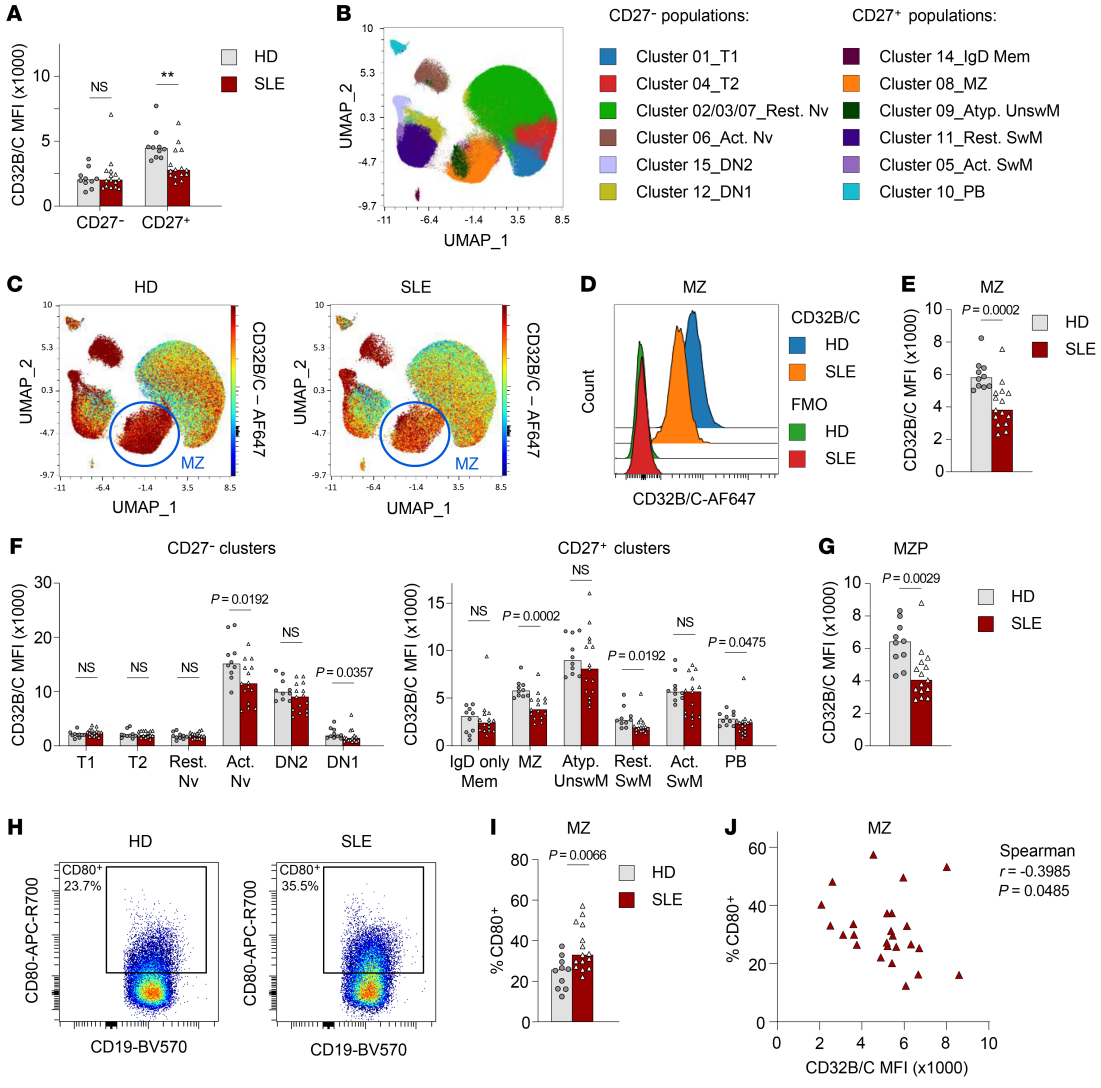
Reduced FcγRIIB expression in MZ B cells from patients with SLE. High-dimensional spectral flow cytometry was used to identify multiple B cell subsets within PBMCs from patients with SLE (*n =* 15) and healthy donors (HD) (*n =* 10). (**A**) Expression of CD32B/C in CD27^–^ and CD27^+^ B cells. (**B**) Live B cells were clustered using FlowSOM and are shown in a UMAP plot. Live B cells from healthy donors and patients with SLE were concatenated from each donor (*n* = 250,000 cells per group). (**C**) Expression of CD32B/C among all B cell clusters in the UMAP plot. (**D**) Representative example of CD32B/C expression in MZ-like B cells from healthy donors and patients with SLE, including the FMO control. (**E**) Summary of CD32B/C expression in MZ B cells. (**F**) Summary of CD32B/C expression in cells in all FlowSOM clusters. (**G**) Expression of CD32B/C in MZP cells identified by manual gating as live CD19^+^CD27^–^IgD^+^CD38^lo^CD21^+^CD24^hi^. (**H**) Representative examples of CD80 expression and gating in MZ B cells from healthy donors and patients with SLE. MZ B cells from healthy donors and patients with SLE were concatenated from each donor (*n* = 25,000 cells per group). (**I**) Summary of the percentage of CD80^+^ MZ B cells from healthy donors and patients with SLE. (**J**) Correlation of the percentage of CD80^+^ cells and CD32B/C MFI in MZ B cells. Data are shown as the median, with each symbol representing an individual donor (*n =* 10 for healthy donors; *n =* 15 for patients with SLE). **P < 0.01 (**A**) and other *P* values were determined by Mann-Whitney *U* test (**E**–**G** and **I**) or Spearman’s rank test (**J**). Act. Nv, activated naive; Rest. Nv, resting naive; Atyp. UnswM, atypical unswitched memory; Act. SwM, activated switched memory; Rest. SwM, resting switched memory; PB, plasmablast.

In line with previous studies, patients with SLE showed decreased expression of CD32B/C on CD27^+^ B cells, but not on CD27^–^ B cells (Figure 6A). Next, B cell subsets were clustered using FlowSOM and visualized on a uniform manifold approximation and projection (UMAP) projection (Figure 6B). MZ B cells were identified in cluster 08, characterized as CD27^+^, IgM^hi^, IgD^lo^, CD21^hi^, CD24^hi^, and CD38^lo^ B cells (Supplemental Figure 10). Likewise, other B cell subsets were identified according to published characteristics (40). Interestingly, we observed a significant decrease in CD32B/C expression in MZ-like B cells (Figure 6, C–E). Although some other cell populations (activated naive B cells, DN1 B cells, resting switched memory B cells, and plasmablasts) also exhibited some decrease in expression of CD32B/C, this difference was no longer significant after correction for multiple testing (Figure 6F). Similar results were obtained using conventional gating (data not shown).

Interestingly, decreased expression of CD32B/C was already observed in the MZ precursor (MZP) population, gated as CD27^–^CD38^lo^IgD^+^CD24^hi^CD45RB^hi^, further characterized in a previous report as IgM^hi^CD21^+^CD1c^int^ (37) (Figure 6G and Supplemental Figure 11).

MZ-like B cells also exhibited significantly increased expression of the activation marker CD80 in patients with SLE compared with healthy donors (Figure 6, H and I). The increased expression of CD80 was not seen in other B cell populations (Supplemental Figure 12). No difference in other activation markers (CD40, CD69, CD86, HLA-DR) was observed (data not shown). CD80 expression in MZ-like B cells was inversely correlated to levels of CD32B/C (Figure 6J), suggesting that the lower expression of FcγRIIB was associated with higher activation of MZ B cells in SLE, as we demonstrated in the murine studies

In summary, our results show increased extrafollicular responses in B cell–intrinsic FcγRIIB-deficient mice characterized by a major increase in MZ-derived IgG3 responses. MZ B cells are highly sensitive to inhibition through FcγRIIB, which can be explained by the high expression of FcγRIIB in MZ B cells compared with expression levels in other B cell subsets. High FcγRIIB expression resulted in strong inhibitory signaling through FcγRIIB in MZ B cells, an effect that we observed in both mice and humans. Finally, patients with SLE exhibited a marked decrease in FcγRIIB expression in B cells, most strongly in MZ-like B cells.

## Discussion

In this study, we analyzed B cell tolerance and extrafollicular PC differentiation in B cell–intrinsic FcγRIIB-deficient mice (16). We have identified spontaneous PC differentiation in *Fcgr2b*-cKO mice, leading to increased serum autoantibody levels, in particular of the IgG3 isotype. In addition, *Fcgr2b*-cKO mice showed increased IgG3 responses following immunization, which was MZ B cell dependent. In both mice and humans, MZ B cells had the highest expression of FcγRIIB; this high expression was associated with increased FcγRIIB-mediated inhibitory effects on Erk phosphorylation and calcium signaling in vitro. Importantly, we showed that MZ B cells from patients with SLE had reduced expression of FcγRIIB that was associated with increased expression of the activation marker CD80. Thus, B cell–intrinsic FcγRIIB deficiency was linked to increased extrafollicular responses through an effect specifically on MZ B cells. In patients with SLE, reduced expression of FcγRIIB may contribute to increased activation of MZ B cells and subsequent autoantibody production.

Similar to previous studies of lupus-prone mice performed in our laboratory (31), we found that B cell deficiency of FcγRIIB led to an increase in PCs, without an increase in the fraction of ANA^+^ IgG PCs, suggestive of increased B cell activation and PC differentiation rather than an antigen-specific tolerance defect. This is also in line with the increased responses to immunization we observed with NP-Ficoll and NP-CGG in *Fcgr2b*-cKO mice. Of note, these mice did not develop a fulminant lupus phenotype, despite the presence of autoreactive PCs in the spleen and autoantibodies in serum. Complete *Fcgr2b*-KO mice on the C57BL/6 background spontaneously develop autoantibody titers and develop a fulminant lupus phenotype (11, 16), probably due to combined effects on B cells and myeloid cells (41).

In contrast to studies with the full FcγRIIB KO (24), we did not observe an effect of B cell–intrinsic FcγRIIB deficiency on B-1 numbers in the spleen or peritoneum, and FcγRIIB expression in B-1 cells was low, suggesting that the effect on the B-1 compartment was indirect. We now show that the enhancement of NP-Ficoll responses in *Fcgr2b*-cKO mice was completely reversed in *Notch2* *Fcgr2b*–deficient dKO mice. Since these mice had largely reduced MZ numbers without significant reductions in B-1 cells (39), this suggests that the enhanced extrafollicular responses to NP-Ficoll were dependent on MZ B cells.

As has been reported in the literature, we observed increased BCR-mediated activation of MZ B cells compared with activation of FO or naive B cells, in both humans and mice (42). Importantly, we showed that MZ B cells also had stronger inhibition mediated by FcγRIIB coengagement. In the MZ B cell compartment, FcγRIIB-mediated inhibition was linked to diminished Erk phosphorylation and calcium flux. Previous studies into the effect of FcγRIIB-BCR crosslinking on B cell signaling have revealed quite variable effects, probably depending on the source and B cell subset studied. Most studies have shown no effect on Syk phosphorylation (43, 44). FcγRIIB engagement preferentially recruits SHIP, which acts to hydrolyze phosphatidylinositol(3,4,5)P3 and inositol(1,3,4,5)P4, downstream of Syk (45). FcγRIIB-BCR crosslinking also leads to earlier closing of calcium channels (44, 46), consistent with our data showing diminished calcium flux. Since we were interested in the difference between MZ B cells and FO B cells, we used primary B cells as opposed to the cell lines that were used in most of the previous studies. Our results show the importance of analyzing signaling in a cell type–specific manner, as different B cell subsets can respond quite differently, and with our studies, we were able to reveal strong inhibitory signaling of FcγRIIB in MZ B cells.

*FCGR2B* risk alleles, which lead to diminished expression and/or inhibitory function of FcγRIIB, predispose individuals to SLE. Furthermore, diminished expression of FcγRIIB on CD27^+^ B cells and plasmablasts from patients with SLE has been shown irrespective of the presence of risk alleles (9, 10). In these studies, the expression of FcγRIIB was studied only in major B cell populations, and IgM^+^ and IgG^+^CD27^+^ B cells were not distinguished. Here, using high-dimension spectral flow cytometry, we were able to show that this difference was mainly driven by reduced expression in MZ B cells. Furthermore, this difference was already present in circulating MZP B cells (37), suggesting that the dysregulation of CD32B/C expression in MZ B cells may occur during early B cell differentiation in SLE. We and others have recently demonstrated that most patients with SLE exhibit enhanced PC differentiation and that some exhibit increased extrafollicular PC differentiation (28, 30, 47). Our current data show how diminished expression of FcγRIIB can lead to increased extrafollicular B cell activation and autoantibody production. Since MZ B cells are known to have increased autoreactivity (31, 48–50), a loss in the regulation of extrafollicular MZ B cell responses through diminished function of FcγRIIB may therefore lead to autoantibody production in SLE.

In summary, we present a model in which high expression of FcγRIIB in MZ B cells was necessary to prevent ongoing activation and thereby functioned as a feedback loop to prevent ensuing autoimmunity, in particular through its effect on extrafollicular MZ responses. As SLE risk alleles for *FCGR2B* diminish the functionality of FcγRIIB, patients with SLE with a risk allele for *FCGR2B* may have a greater impairment of MZ B cells and may have the highest levels of MZ-derived autoantibodies and the largest fluctuation in autoantibody titers. We believe these studies move us closer to precision medicine in SLE.

## Methods

### Mice.

*Fcgr2b^fl/fl^* mice were a gift from Jeffrey Ravetch (The Rockefeller University, New York, New York, USA) (16). CD19^Cre^ mice (stock no. 006785), B6.C-H2d (stock no. 000359) were obtained from The Jackson Laboratory and were crossed with *Fcgr2b^fl/fl^* mice to generate CD19^Cre/+^ *Fcgr2b^fl/fl^* H2d/d (*Fcgr2b*-cKO) mice. Control mice were either carrying only the Cre allele (CD19^Cre/+^ *Fcgr2b^WT/WT^* H2d/d) or only the floxed alleles (CD19^+/+^ *Fcgr2b^fl/fl^* H2d/d). To generate MZ-deficient mice, CD19^Cre/+^ control mice and *Fcgr2b*-cKO mice were crossed with *Notch2^fl/fl^* mice (The Jackson Laboratory, stock no. 010525) to generate CD19^Cre/+^ *Notch2^fl/fl^* H2d/d and CD19^Cre/+^ *Fcgr2b^fl/fl^* *Notch2^fl/fl^* H2d/d mice.

For analysis of spontaneous autoimmunity, mice were kept until 10–12 months of age. For analysis of immunization responses, 8- to 16-week-old female mice were immunized i.p. with 50 μg NP-Ficoll (conjugation ratio, 55:1) in 100 μL saline (Biosearch Technologies) or 100 μg NP-CGG (conjugation ratio, 20–29:1) (Biosearch Technologies) in Imject Alum (Thermo Fisher Scientific) and followed for 7 days (both NP-Ficoll and NP-CGG) and for 42 days (NP-CGG only). For functional in vitro studies of FcγRIIB, spleens of 8- to 16-week-old female mice were used.

Spleens and BM were collected at the indicated time points followed by the formation of a single-cell suspension by mashing over a 70 μm cell strainer. RBC lysis was performed using RBC lysis buffer (BioLegend). Peritoneal cells were obtained by injecting 10 mL HBSS plus 5% FBS, followed by massaging of the peritoneum and withdrawal of the buffer with cells. Serum was obtained through submandibular bleeding followed by centrifugation.

### Patients and healthy donors.

Buffy coats from healthy donors were obtained through the Sanquin blood bank (Netherlands). PBMCs were isolated using the standard Ficoll procedure. For signaling experiments, frozen PBMCs obtained from buffy coats were used.

For spectral flow cytometry, heparinized blood was obtained from patients with SLE (*n =* 15) and healthy donors (*n =* 10). Patients with SLE were recruited from the Rheumatology outpatient clinic of the Leiden University Medical Center in the Netherlands, and healthy donor samples were obtained through the LUMC Voluntary Donor Service Biobank (LuVDS, Leiden University Medical Center, Leiden, Netherlands). The age and sex of the healthy donors were selected to reflect the sex and age range of the patients with SLE. PBMCs were isolated using a standard Ficoll procedure and frozen.

### ELISA.

Half-area ELISA Plates (Corning) were coated with 10 μg/mL of NP2- or NP25-BSA (Biosearch technologies) and anti–mouse IgG, IgM, IgG1, IgG2b, IgG2c, or IgG3 unlabeled antibody (Southern Biotech) overnight at 4°C, or with 100–400 μg/mL sonicated filtered calf thymus DNA (Calbiochem) overnight at 37°C uncovered. Plates were washed with PBS containing 0.05% Tween-20 and blocked with 1% BSA in PBS. Diluted serum samples were incubated for 1.5 hours. Serum samples for anti-DNA IgG and IgG subclasses were diluted 1:100. Serum samples from NP-immunized mice were diluted 1:10,000 (day 7) or 1:50,000 (time course days 0, 14, 28, 42) for NP-specific IgM and IgG, and 1:2500 for NP-specific IgG subclasses. Plates were washed with wash buffer and secondary goat polyclonal alkaline phosphatase–labeled (AP-labeled) anti–mouse IgG, IgM, IgG1, IgG2b, IgG2c, or IgG3 (Southern Biotech) was added for 1 hour. After washing, plates were developed using phosphatase substrate (MilliporeSigma) dissolved in distilled water with 50 mM NaHCO_3_ (MilliporeSigma) and 1 mM MgCl_2_ (MilliporeSigma). Plates were read at a wavelength of 405 nM on a 1430 Multilabel Counter Spectrometer (PerkinElmer).

### Flow cytometry.

For flow cytometry phenotyping, cells were preincubated on ice for 5 minutes with Fc block (anti-mouse or anti-human) for each staining, except the staining for FcγRIIB. After this, cells were stained for 30 minutes with antibodies against cell membrane proteins followed by washing in 5% FBS in HBSS or 0.5% BSA in PBS. For surface staining, cells were washed and stored in 1% paraformaldehyde (PFA) until acquisition. For intracellular staining, cells were fixed and permeabilized with a Foxp3 transcription factor fixation/permeabilization kit (eBioscience) for 45 minutes on ice. After fixation and permeabilization, the intracellular staining cocktail was incubated for 30 minutes in permeabilization buffer to visualize immunoglobulins and ANA or NP reactivity in PCs.

The antibodies used are listed in Supplemental Table 1. For staining of NP-specific B cells in immunized mice, NP(6)-BSA-biotin (Biosearch Technologies) was preincubated for 30 minutes with streptavidin-APC at a 1:1 molar ratio, after which cells were incubated with 0.1 μg/mL NP-BSA-biotin^+^ streptavidin-APC during either the surface or intracellular staining step. Surface and intracellular ANA stainings were performed as described previously (31).

Mouse gating strategies were based on our prior studies (28, 31). Live cells were gated using DAPI or fixable viability dye eF506. B cell subsets in mouse BM were gated as immature B cells: B220^+^CD43^–^CD24^hi^CD21^lo^IgD^lo^IgM^hi^; emigrating B cells: B220^+^CD43^–^CD24^hi^CD21^lo^IgD^hi^IgM^hi^; and recirculating B cells: B220^+^CD43^–^CD24^+^CD21^+^IgM^hi^IgD^hi^. B cell subsets in mouse spleen were: T1: B220^hi^CD93^+^CD23^lo^IgM^hi^; T2: B220^hi^CD93^+^CD23^hi^IgM^hi^; T3 B cells: B220^hi^CD93^+^CD23^hi^IgM^lo^; FO: B220^hi^CD93^–^CD21^lo^ with either IgD^hi^, CD23^+^, or both; and MZ: B220^hi^CD93^–^CD21^hi^ with either IgD^lo^, CD23^lo^, or both. B1 B cells in spleen and peritoneum were gated as: B220^lo^CD19^hi^CD93^–^CD21^–^IgD^lo^, with CD5 used to separate B1a and B1b cells. Memory B cells were gated as Ig^D–^CD38^+^ GL7, activated/pre-GC B cells were gated as IgD^–^CD38^+^GL7^+^, and GC B cells were gated as IgD^–^CD38^–^GL7^hi^. CD95 expression on GC B cells was used to further confirm the GC gating. PCs were gated on the basis of B220^lo/+^ and CD138^hi^ expression and were validated by intracellular Ig staining. IgG subclasses and specificity (ANA/NP) were determined within memory, activated, GC B cells and PC populations. For Phosphoflow experiments, staining for CD93, CD21, and CD23 was performed prior to activation (due to loss of signal following fixation/permeabilization), while all other antibodies were included in the intracellular staining mix.

For human B cell gating strategies, all cells were gated as live on the basis of Fixable Viability Dye eFluor 506 (Thermo Fisher Scientific) labeling. Naive B cells were gated as CD19^+^CD27^–^CD38^lo^IgG^–^IgA^–^IgD^hi^IgM^lo^; and MZ-like B cells were gated as CD19^+^CD27^+^CD38^lo^IgG^–^IgA^–^IgD^lo^IgM^hi^. For functional assays, staining with IgM/IgD needed to be avoided to prevent activation of cells through their BCR, but their phenotype was confirmed by subsequent staining for IgM/IgD. For Phosphoflow experiments, CD27 staining was performed prior to activation (due to loss of signal following fixation/permeabilization), while all other antibodies were included in the intracellular staining mix.

### In vitro B cell activation.

For mouse in vitro activation studies, FO B cells and MZ B cells were sorted using the FACSAria or FACSAria SORP (BD). For dead cell exclusion, 20 ng/mL DAPI (Thermo Fisher Scientific) was added to the cells just prior to sorting. FO B cells and MZ B cells were cultured in RPMI medium plus penicillin-streptomycin, l-glutamine, and 10% FBS and stimulated with polyclonal goat anti–mouse IgM (Southern Biotech; 0.6–150 μg/mL), or with equimolar concentrations of the Fab′2 fragments (Southern Biotech; 0.4–100 μg/mL) for 20 hours, after which activation was measured using flow cytometry as described above.

For human in vitro activation studies, naive and MZ-like B cells were sorted using the BD FACSAria system. Cells were cultured in RPMI medium plus penicillin-streptomycin, l-glutamine, and 10% FBS and stimulated with polyclonal goat anti–human IgM (Jackson ImmunoResearch, AffiniPure; 0.6–75 μg/mL), or with equimolar concentrations of the Fab′2 fragments (Jackson Immunoresearch Affinipure; 0.4–50 μg/mL) for 20 hours, after which activation was measured using flow cytometry as described above.

### B cell signaling.

For Phosphoflow experiments, mouse splenocytes or human PBMCs were stimulated for 2, 10, and 60 minutes in RPMI medium, penicillin-streptomycin, l-glutamine, and 10% FBS with the anti-IgM antibodies described above (intact 3–15 μg/mL; Fab′2, 2–10 μg/mL). Cells were immediately fixed in 1× Phosphoflow Lyse/Fix buffer (BD). After 10–12 minutes of incubation at 37°C, cells were washed and permeabilized using Phosphoflow Perm Wash I (BD) for 30–60 minutes. Following a 5-minute preincubation with Fc block (BD), cells were then incubated with a mix of antibodies against cell-surface and phospho-specific signaling molecules or isotype controls at room temperature for 60 minutes.

For capillary Western blotting, mouse FO B cells and MZ B cells were sorted and stimulated for 10 minutes with the anti-IgM antibodies (intact 7.5 μg/mL; Fab′2, 5 μg/mL) in the supplemented RPMI medium described above. Stimulation was immediately ceased by adding ice-cold PBS. Cells were lysed in Cell Lysis buffer (BD) supplemented with Halt Protease and Phosphatase Inhibitor Cocktail (Thermo Fisher Scientific) at 1 × 10^5^ cells/3 μL. After 1 hour of incubation on ice, the cell lysate was collected and stored frozen.

Capillary Western blotting was performed with Jess (Protein Simple) according to the manufacturer’s instructions. Briefly, cell lysates from 1 × 10^5^ cells were loaded into each capillary. SHIP1 was detected with rabbit anti–mouse SHIP1 antibody (Cell Signaling Technology, polyclonal rabbit; catalog 2728; diluted at 1:10), followed by antibody stripping and p-SHIP1 (Tyr1020) detection with rabbit anti–mouse p-SHIP1 antibody (Cell Signaling Technology, polyclonal rabbit, catalog 3941; diluted at 1:10). The data obtained were analyzed with Compass for SW software (Protein Simple). The AUC of the band intensity histograms was used to calculate the amount of protein.

For calcium flux experiments, human PBMCs were labeled with 0.5 μM Indo-1 AM (Thermo Fisher Scientific) in the presence of 0.02% Pluronic F-127 (Thermo Fisher Scientific) in IMDM plus 2% FBS, for 45 minutes at 37°C, after which cells were washed twice, followed by staining of surface markers on ice. After washing, cells were taken up in colorless IMDM plus 2% FBS. Cells and stimuli were prewarmed at 37°C for a minimum of 10 minutes before measuring. Calcium flux measurements were performed on a BD LSR-II. After 30 seconds of establishment of baseline Indo-1 signals, cells were stimulated with the anti-IgM antibodies described above (intact 15–75 μg/mL; Fab′2, 10–50 μg/mL), after which acquisition was immediately resumed and continued for 2.5 minutes. Ionomycin (MilliporeSigma) was used at 1 μg/mL as a positive control.

### RNA isolation and quantitative PCR.

Human naive, MZ-like, and conventional memory (IgG^+^ and IgA^+^) B cells were sorted according to the gating strategy described above. A total of 50,000–200,000 cells per population were lysed in RLT buffer (QIAGEN) with 10 μL/mL 2-ME (Merck), followed by RNA isolation using the RNeasy Microprep kit (QIAGEN). cDNA was synthesized using iScript (Bio-Rad). Quantitative PCR (qPCR) was performed after preamplification using TaqMan PreAmp Master Mix (Thermo Fisher Scientific) and the TaqMan assays mentioned below. qPCR was performed using TaqMan Fast Advanced Master Mix (Thermo Fisher Scientific) and the following multiplexed VIC and FAM TaqMan assays (all from Applied Biosystems/Thermo): *POLR2A* VIC-MGB (Hs00172187_m1); *ACTB* VIC-MGB (Hs01060665_g1); *HOPX* FAM-MGB (Hs04188695_m1); *SOX7* FAM-MGB (Hs00846731_s1); and *FCGR2B* FAM-MGB (Hs00269610_m1). qPCR was run on a CFX Opus machine (Bio-Rad) using the recommended cycling conditions.

### Spectral flow cytometry and clustering analysis.

For spectral flow cytometry, cells were stained as described for conventional flow cytometry using with the antibodies listed in Supplemental Table 2. Antibody cocktails were prepared using Brilliant Stain Buffer (BSB) Plus (BD). Monocyte blocker (BioLegend) was added to prevent aspecific binding of tandem dyes. To determine the specificity of CD32B/C staining, a fluorescence minus one (FMO) control was taken along for each sample. Cells were washed and fixed with 1% PFA for 20 minutes. Cells were washed again and stored in FACS buffer until acquisition on a 5-laser Aurora (Cytek). Raw spectral data were unmixed using SpectroFlo software (Cytek), after which unmixed fcs files were analyzed in OMIQ software. Live B cells were gated, after which B cell populations were gated manually, and expression levels of CD32B/C were obtained. In parallel, live B cells were clustered with FlowSOM (elbow metaclustering) using CD19, CD20, CD21, CD24, CD27, CD38, IgD, and IgM as input parameters. Using the same parameters, cells were projected onto UMAP (neighbors = 15, mindist = 0.2). Expression levels of markers were analyzed in UMAP and heatmaps to identify known B cell subsets (40). Fifteen FlowSOM metaclusters were obtained, among which 3 clusters were very similar in expression patterns and were together designated as resting naive. One small cluster (<1%) was identified as a non–B cell cluster and excluded from downstream analysis, leaving 12 final B cell clusters.

### Data analysis.

Analysis of flow cytometric data was performed using the BD FACS DIVA and FlowJo software. Graphing of calcium flux was performed using GraphPad Prism (GraphPad Software) after exporting the binned data for each population to FlowJo. Cells were gated as percentage positive if there were 2 clear populations, whereas median fluorescence intensity (MFI) was used when an entire population showed a shift. All intensities are shown as MFIs. To control for differences in autofluorescent backgrounds or nonspecific binding, isotype controls were used for in vitro activation and Phosphoflow experiments. No differences in isotype control background were found between the cell populations that were compared. In vitro activation and phosphorylation data are shown for 3 μg/mL intact and 2 μg/mL Fab′2 anti-IgM antibodies. For both mouse and human studies, similar results were obtained with 10 and 15 μg/mL, respectively (data not shown). For in vitro activation and signaling, the percentage of inhibition with intact anti-IgM was calculated per donor as follows: intact anti-IgM/(Fab′2 anti-IgM – unstimulated) × 100.

Immunized mice were excluded when they exhibited no serum antibody response compared with baseline (*n =* 1 for timeline NP-CGG response; *n =* 3 for the day-7 NP-CGG response; *n =* 1 for the day-7 NP-Ficoll response). One sample for FcγRIIB expression in human B cells was excluded from the qPCR analysis because it was an extreme outlier of protein expression in naive B cells (Figure 3I). Serum samples were excluded because they were extreme outliers (>mean + 4 × SD; *n =* 3 excluded) in the Notch2 experiments for spontaneous autoantibody production (Figure 5, G–I).

### Statistics.

Statistical analysis was performed using GraphPad Prism (GraphPad Software). For comparison of ex vivo mouse data with 2 groups, a 2-tailed Mann-Whitney *U* test was performed. For comparison of multiple groups, 1-way ANOVA with Bonferroni’s post hoc test was used. For 2-group analysis of in vitro activation, a 2-tailed, paired *t* test was used, and for comparison of 2 categorical variables, 2-way ANOVA with Bonferroni’s post hoc test was used. Comparison of expression levels in healthy donors versus patients with SLE was performed using a 2-tailed Mann-Whitney *U* test. Correlation was calculated using Spearman’s rank test. *P* values of less than 0.05 were considered statistically significant.

### Study approval.

Mice were housed according to Association for Assessment and Accreditation of Laboratory Animal Care (International) (AAALAC) regulations, and all mouse studies were approved the IACUC of The Feinstein Institutes for Medical Research/Northwell health. Studies with buffy coats from healthy donors were performed in accordance with the Declaration of Helsinki, and written informed consent was obtained from all donors. Studies of blood from patients with SLE and healthy donors were approved by the local medical ethics committee (METC-LDD, Leiden, The Hague, Delft, Netherlands), and written informed consent was obtained from all donors.

## Author contributions

ANB designed and conducted experiments, analyzed data, and wrote the initial manuscript. SM designed and conducted experiments and analyzed data and revised the manuscript. IKS and ALD conducted experiments and analyzed data. JS conceptualized the study, designed and conducted experiments, analyzed data, and wrote the manuscript. BD conceptualized the study, designed experiments, interpreted the data, and wrote the manuscript. The order of the co–first authors was determined by who initiated the study (ANB) and who completed the study (SM).

## Supplementary Material

Supplemental data

## Version 1 07/12/2022

In-Press Preview

## Version 2 09/01/2022

Electronic publication
