# Supplementary material for: FcγRIIB regulates autoantibody responses by limiting marginal zone B cell activation
Source: J Clin Invest. 2022 Sep 1;132(17):e157250. doi: 10.1172/JCI157250 (PMC9435648; doi:10.1172/JCI157250)
Supplement: Supplemental data [file jci-132-157250-s131.pdf]

Supplementary file (Figure S1-12, Table S1-S3)

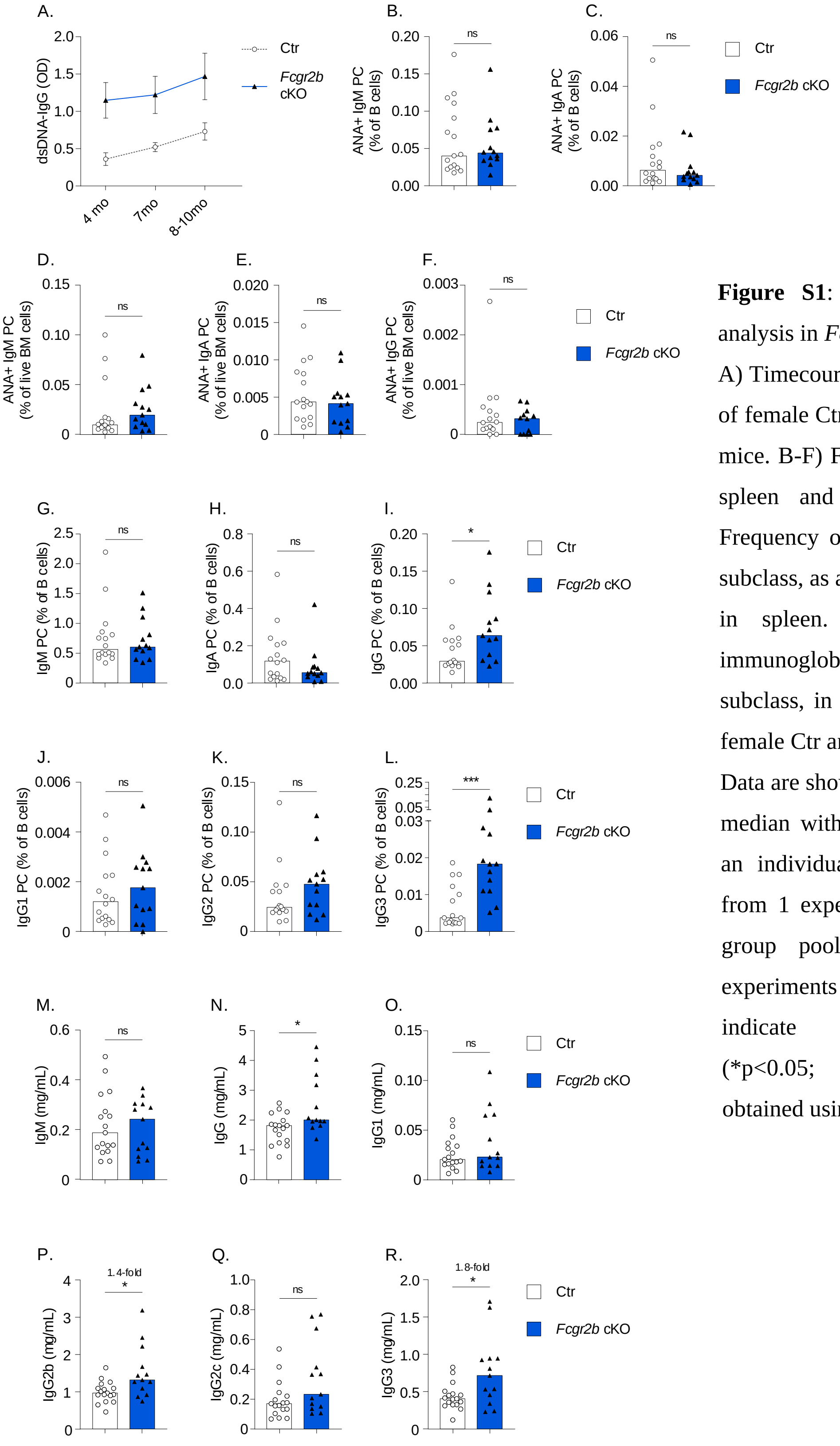

Figure S1: Detailed serum and PC analysis in *Fcgr2b* cKO mice

A) Timecourse of dsDNA IgG on serum of female Ctr and *Fcgr2b* cKO deficient mice. B-F) Frequency of ANA+ PCs in spleen and BM, by isotype. G-L) Frequency of PCs by isotype and IgG subclass, as a percent out of total B cells in spleen. M-R) Levels of total immunoglobulin, by isotype and IgG subclass, in serum of 10-12 month old female Ctr and *Fcgr2b* cKO mice.

Data are shown as mean +/- SEM (A) or median with each symbol representing an individual mouse (n=7 per group from 1 experiment in A; n=13-17 per group pooled from 3 independent experiments in other panels). Asterisks indicate significant differences (*p<0.05; **p<0.01; ***p<0.001) obtained using Mann whitney U test.

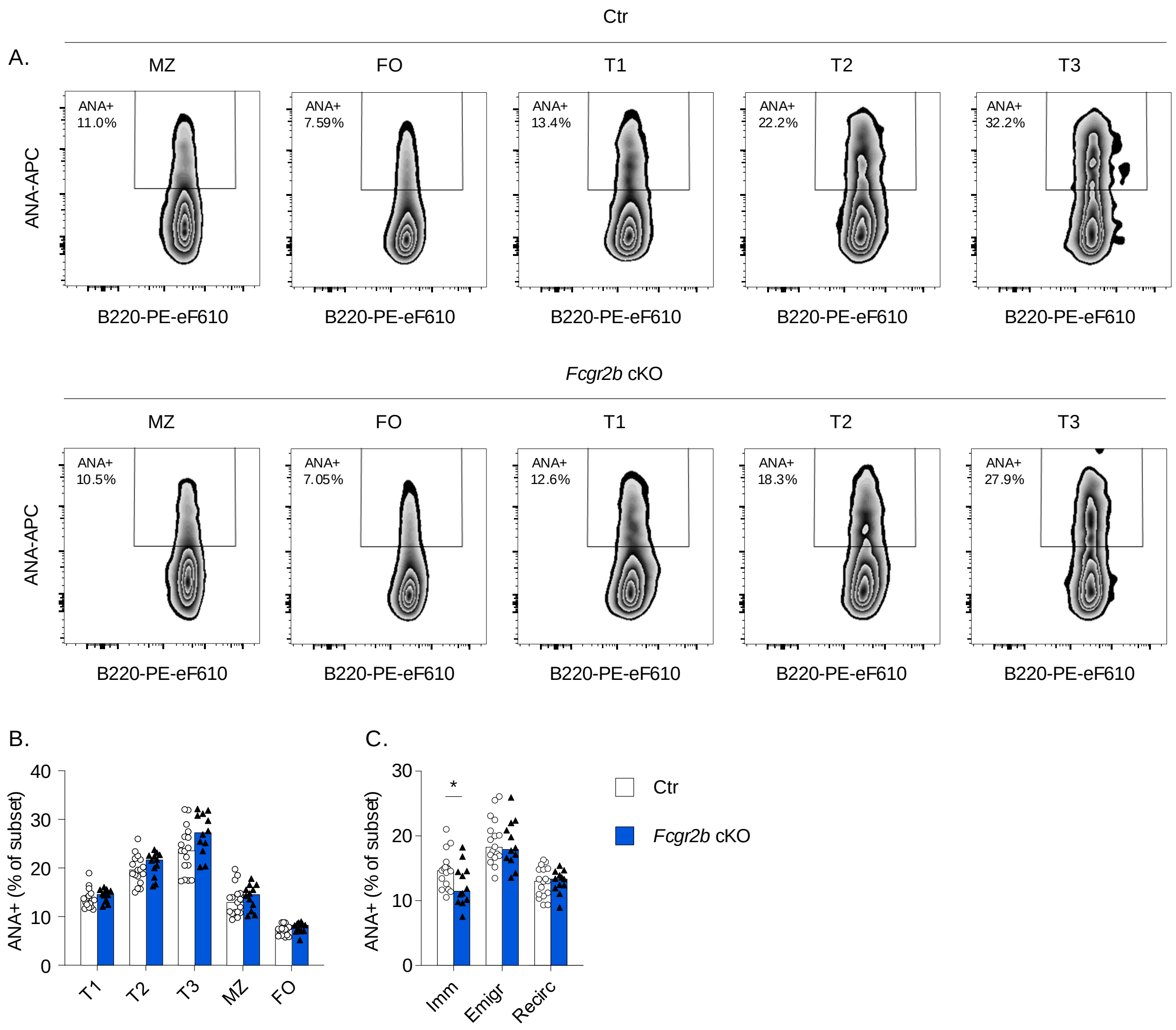

Figure S2: Early tolerance checkpoints for ANA in BM and spleen.

The frequency of autoreactive ANA+ B cells in 10-12 month old control (Ctr) and *Fcgr2b* cKO mice was established using flow cytometry through ANA surface staining. A) Representative example of ANA staining in B cell subsets in the spleen. B-C) Summary of ANA reactivity in B cell subsets in the spleen and bone marrow. B cell subsets were gated as described in Methods.

Data are shown as median with each symbol representing an individual mouse (n=12-17 per group pooled from 3 independent experiments). Asterisks indicate significant differences (*p<0.05; **p<0.01; ***p<0.001) obtained using Mann whitney U test.

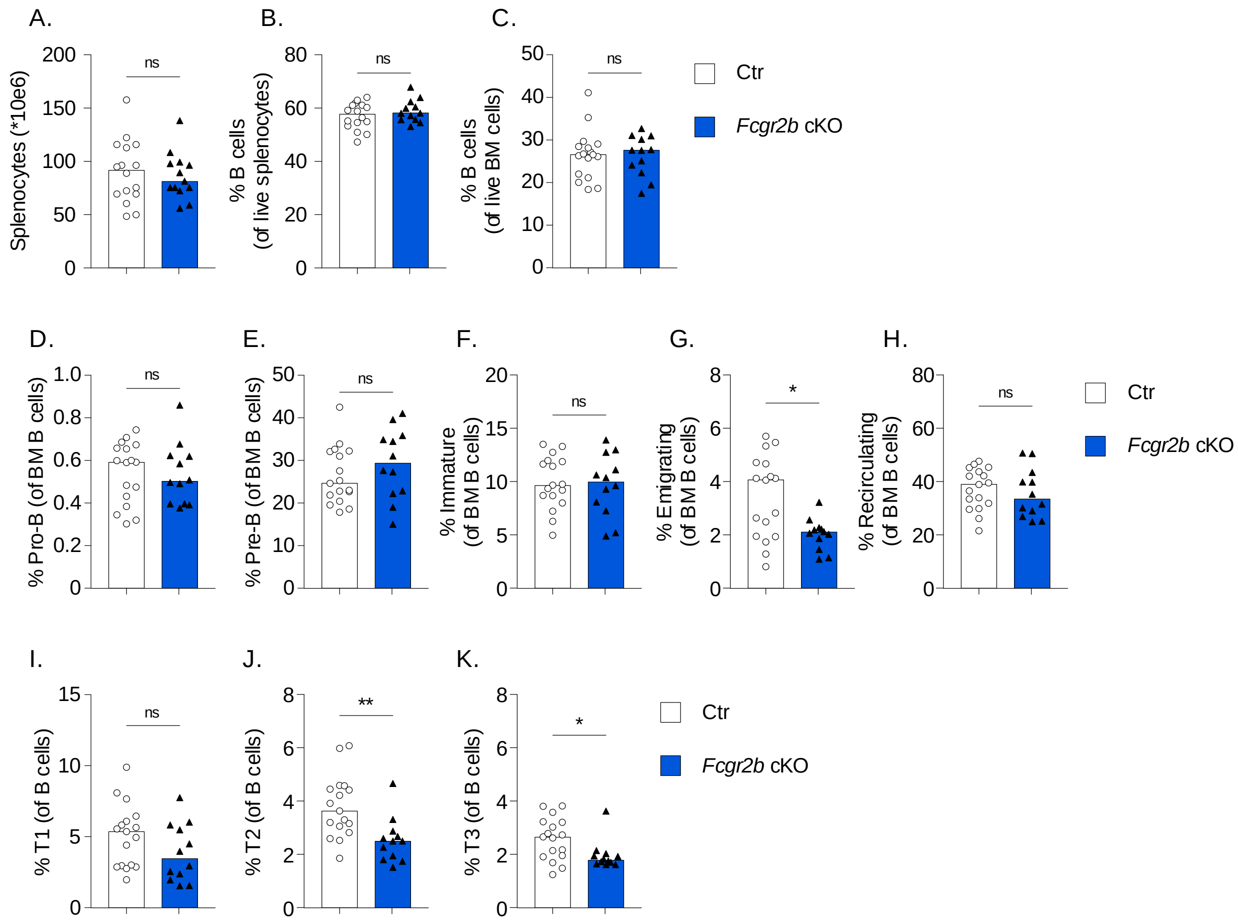

Figure S3: B cell subsets in BM and spleen.

The frequency of B cell subsets in spleen and bone marrow of 10-12 month old control (Ctr) and *Fcgr2b* cKO mice was established using flow cytometry. A) Total splenocyte count. B,C) Frequency of total B220+ B cells in spleen and bone marrow. Representative example of ANA staining in B cell subsets in the spleen. C-M) Summary of B cell subset frequencies in the spleen and bone marrow. B cell subsets were gated as described in Methods.

Data are shown as median with each symbol representing an individual mouse (n=12-17 per group pooled from 3 independent experiments). Asterisks indicate significant differences (*p<0.05; **p<0.01) obtained using Mann whitney U test.

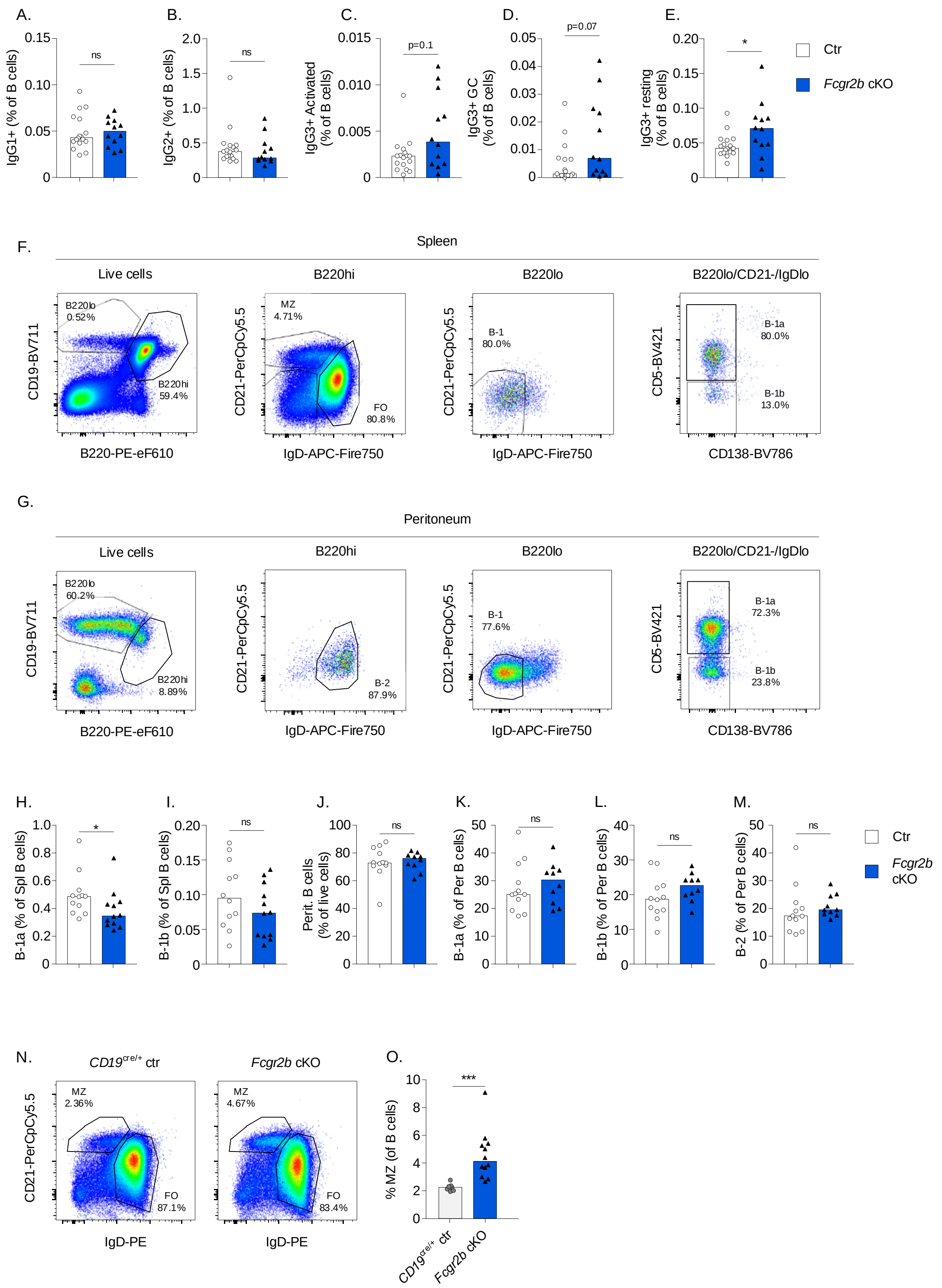

Figure S4: IgG class-switched cells, B-1 and MZ B cells in *Fcgr2b* cKO mice.

A,B) Frequency of IgG1+ and IgG2+ B cells in 10-12 month old female control (Ctr) and *Fcgr2b* cKO mice. C-E) Frequency of IgG3+ B cell subsets based on CD38 and GL7 staining. F,G) Representative examples of B cell staining

strategies in spleen and peritoneum. B220hi = B-2 cells and B220o = B-1 cells, further confirmed as CD21loIgD- and separated into B-1a and B-1b based on CD5 (right panel). H-M) Frequency of B-1 cells in spleen (H,I) and total B cells, B-1a, B-1b, and B-2 cells in peritoneum (J-M). N,O) Representative example and summary of MZ frequency within mature CD93- B cells.

Data are shown as median with each symbol representing an individual mouse (n=12-17 per group for A-E; n=10-12 per group for H-M; n=8=12 per group for N-O; each pooled from 2-3 independent experiments). Asterisks indicate significant differences (*p<0.05; **p<0.01; ***p<0.001) obtained using Mann whitney U test.

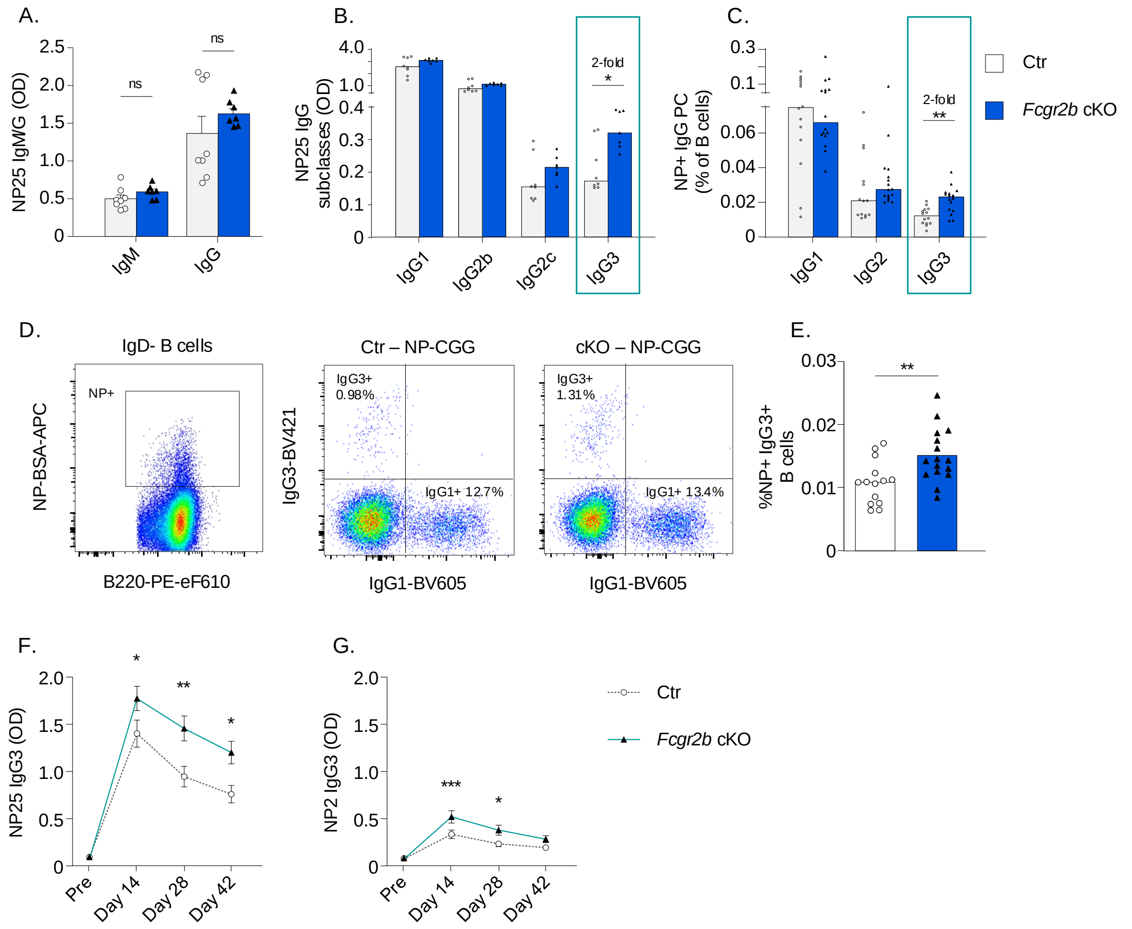

Figure S5: Characterization of T-dependent response to NP-CGG.

Female Ctr and *Fcgr2b* cKO mice were immunized with NP-CGG in Alum. Serum and splenocytes were obtained after 7 days (except F-H). A,B) Levels of NP-specific antibodies, separated by isotype and subclass. C) Frequency of NP-specific PCs in spleen as percentage of B cells, separated by IgG subclass. D) Representative example of surface NP gating on IgD- B cells (left) and IgG1 and IgG3 staining in IgD-NP+ B cells (middle and right). E) Frequency of IgG3+ NP+ B cells out of total B cells. F-G) Timecourse of serum titers for NP25- and NP2-specific IgG3.

Data are shown as median with each symbol representing an individual mouse (n=7-8 per group for A,B; n=14-16 for C,E; n = 17-20 for F-G; each pooled from 2-3 independent experiments). Asterisks indicate significant differences (*p<0.05; **p<0.01; ***p<0.001) obtained using Mann Whitney U test (A-E) or Two-way ANOVA with Bonferroni posthoc test (F-G).

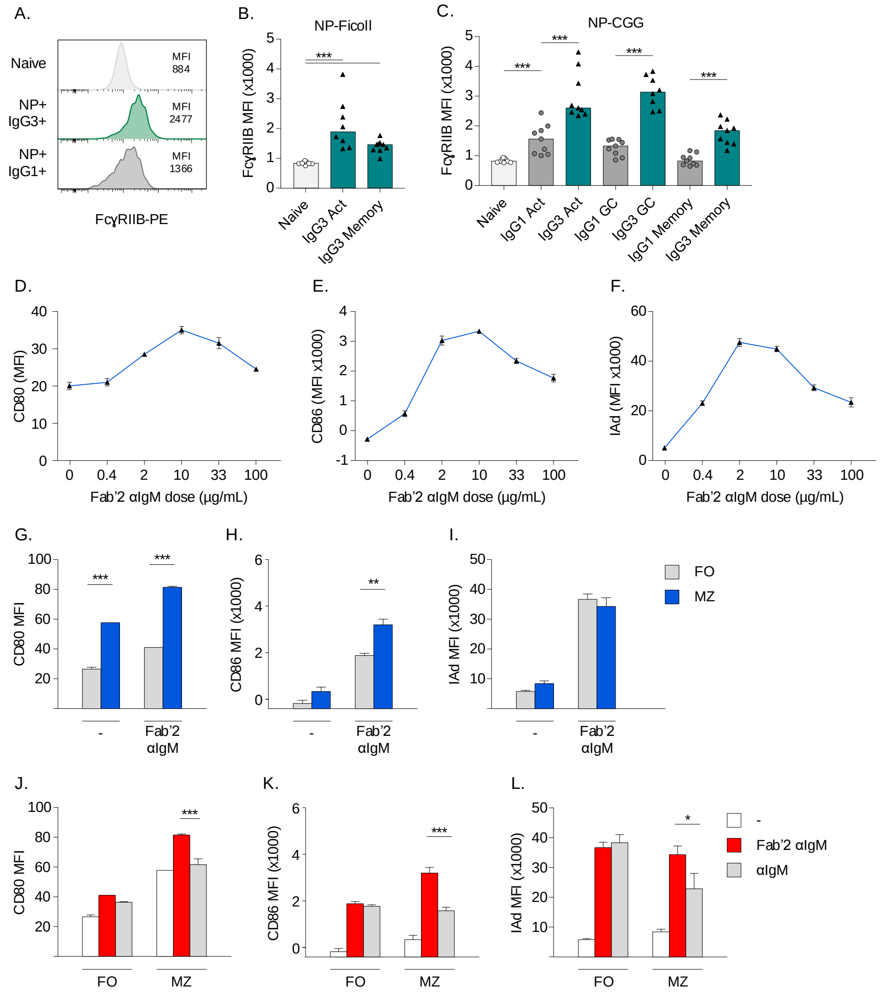

Figure S6: FcγRIIB expression and In vitro activation of follicular (FO) and marginal zone (MZ) B cells using intact and Fab’2 anti-IgM antibodies.

A) Representative example of staining for FcγRIIB in NP+ IgG1+ and IgG3+ B cells in control mice immunized with NP-CGG. B,C) FcγRIIB staining intensity in Naïve B cells, and different subsets of IgG1+ and IgG3+ B cells following immunization with NP-Ficoll and NP-CGG (day 7). D-L) FO and MZ B cells were sorted from the spleens of Ctr and *Fcgr2b* cKO mice, followed by stimulation with intact or equimolar concentrations of Fab’2 anti-IgM for 20 hours. Activation was measured by flow cytometry. D-F) Titration of Fab’2 anti-IgM stimulation in FO B cells. G-I) Comparison of activation of FO and MZ B cells in Ctr mice. J-L) Comparison of intact versus Fab’2 anti-IgM in FO and MZ B cells.

Data are shown as median (B,C) or mean +/- SEM (D-L) (n=8-9 per group for B-C; n=4-7 per group for G-L; each pooled from 2-3 independent experiments; except D-F which was from 1 experiment with 2 mice). Asterisks indicate significant differences (*p<0.05; **p<0.01; ***p<0.001) obtained using Mann Whitney U test (B,C) or Two-way ANOVA with Bonferroni posthoc test (G-L).

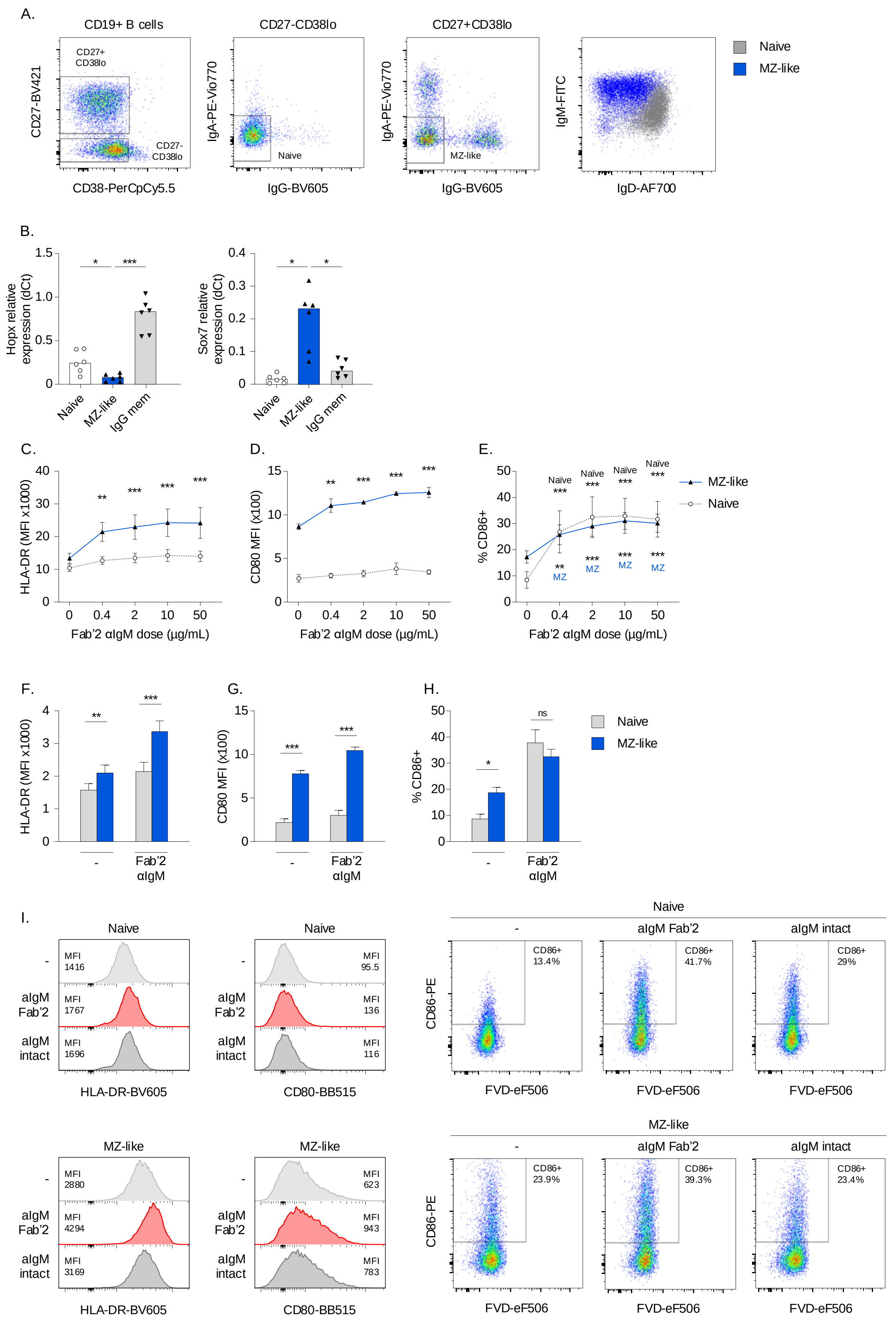
A.
CD19+ B cells
CD27-CD38lo
CD27+CD38lo
CD27+ CD38lo
CD27- CD38lo
Naive
MZ-like
CD27-BV421
CD38-PerCpCy5.5
IgA-PE-Vio770
IgG-BV605
IgM-FITC
IgD-AF700
B.
Hopx relative expression (dCt)
Sox7 relative expression (dCt)
Naive
MZ-like
IgG mem
C.
D.
E.
HLA-DR (MFI x1000)
CD80 MFI (x100)
% CD86+
Fab'2 αIgM dose (µg/mL)
F.
G.
H.
Fab'2 αIgM
I.
Naive
MZ-like
αIgM Fab'2
αIgM intact
HLA-DR-BV605
CD80-BB515
CD86-PE
FVD-eF506

Figure S7: Human MZ-like B cell phenotype, titration of aIgM, and effect of Fab'2 anti-IgM on B cell activation,
A) Gating strategy for human Naive and MZ-like B cells, starting with live CD19+ B cells. Cells were sorted from PBMCs of healthy donors, and analyzed by flow cytometry. B) qPCR for *HOPX* and *SOX7* in sorted B cell subsets, confirming MZ-like phenotype. Relative expression was normalized to polr2a. C-I) sorted Naïve and MZ-like B cells which were stimulated with intact anti-IgM or equimolar concentrations of Fab'2 anti-IgM 20 hours. Activation was measured by flow cytometry. C-E) Titration of Fab'2 anti-IgM in sorted Naïve and MZ-like B cells. F-H) Comparison of Naïve and MZ-like B cell activation, stimulated with 2 ug/mL Fab'2 anti-IgM. I) Representative examples of sorted Naïve and MZ-like B cells which were stimulated with 3 ug/mL intact anti-IgM or equimolar concentrations (2 ug/mL) of Fab'2 anti-IgM 20 hours. Activation was measured by flow cytometry.
Data are shown as median with each symbol representing an individual donor (B) or mean +/- SEM (C-H) (n=4-10 per group from 2-5 independent experiments for B; n=4 per group from 2 independent experiments for C-E; n=10 per group from 5 independent experiments for F-H). Asterisks indicate significant differences (*$p<0.05$; **$p<0.01$; ***$p<0.001$) obtained using Wilcoxon signed rank test (B) or Two-way ANOVA with Bonferroni posthoc test (C-H).

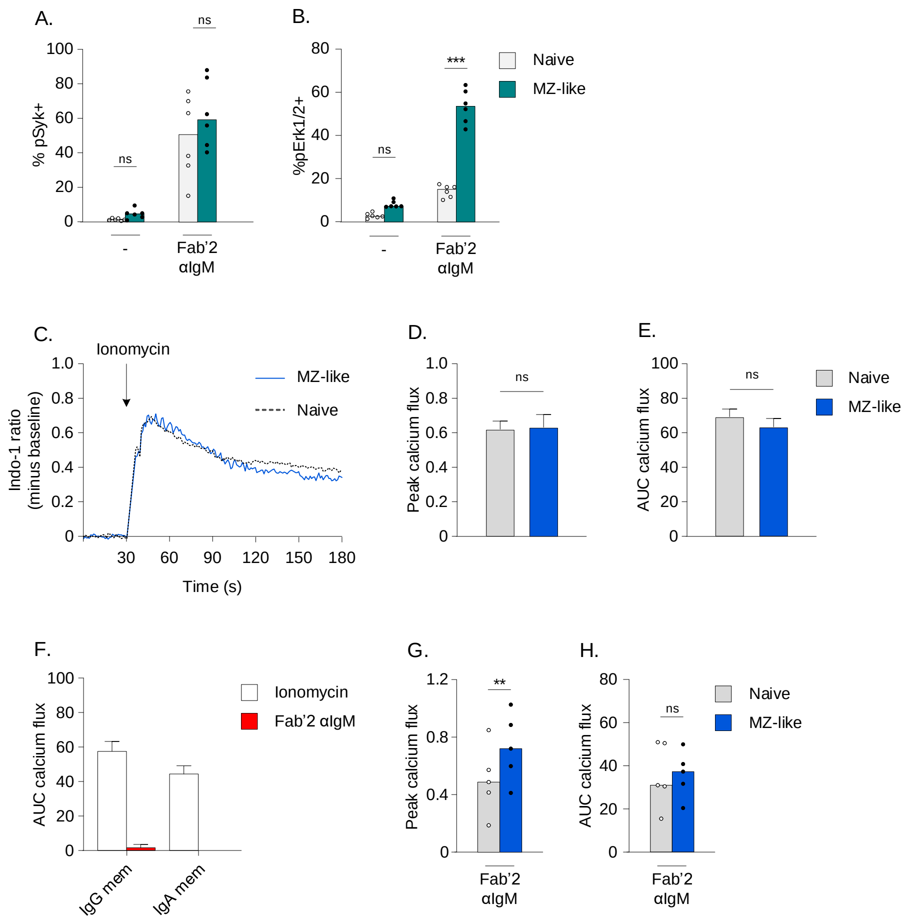

Figure S8: Phosphoflow and calcium flux with Fab'2 anti-IgM.

A,B) PBMCs from healthy donors were stimulated with 10 ug/mL of Fab'2 anti-IgM for 10 min, after which the phosphorylation of signaling molecules was analysed by phosphoflow. Comparison of phosphorylation of Syk and Erk in Naïve versus MZ-like B cells. C-H) PBMCs from healthy donors were labeled with Indo-1 for calcium flux measurements. After 30 seconds of baseline measurement, 75 ug/mL intact anti-IgM or equimolar concentrations (50 ug/mL) of Fab'2 anti-IgM were added and the measurement continued for 2.5 min. 1 ug/mL Ionomycin was used as a positive control. Peak calcium flux and area under the curve (AUC) were calculated using Flowjo. C-E) Representative examples and summary of Ionomycin-induced calcium flux in Naïve and MZ-like B cells gated as in Figure S7A. F) Lack of calcium flux in IgG+ and IgA+ B cells following Fab'2 anti-IgM in comparison to Ionomycin as positive control. G,H) Comparison of the peak and AUC of calcium flux in Naïve and MZ-like B cells following stimulation with Fab'2 anti-IgM.

Data are shown as mean +/- SEM (n=5-6 per group from 2-3 independent experiments). Asterisks indicate significant differences (*p<0.05; **p<0.01; ***p<0.001; Ns: not significant) obtained using Two-way ANOVA with Bonferroni posthoc test (A,B) or Mann Whitney U test (D-H).

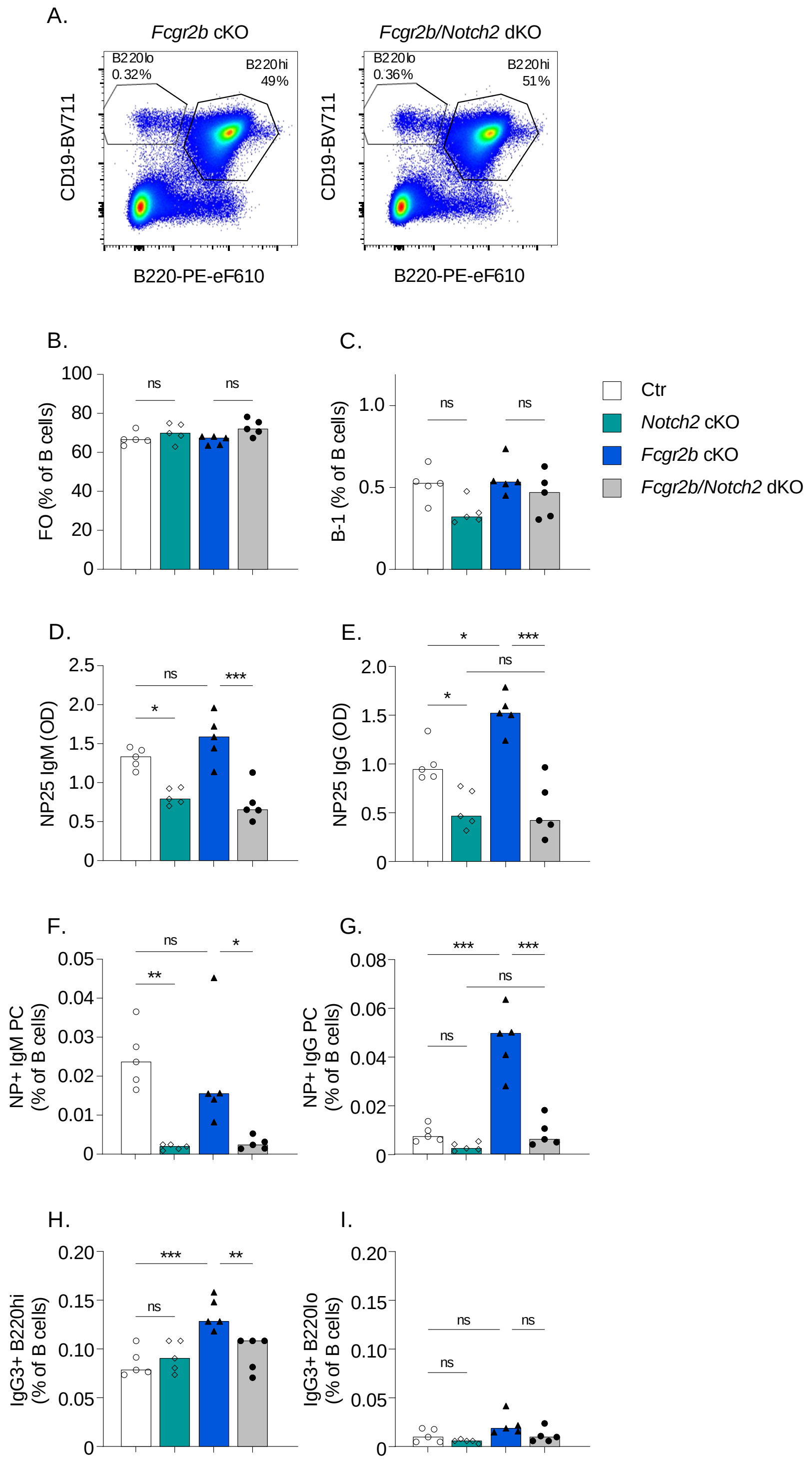

Figure S9: Combined FcɣRIIB and MZ deficiency reverses enhanced extrafollicular responses.

Female Ctr, *Notch2* cKO, *Fcgr2b* cKO, and *Notch2*/*Fcgr2b* dKO mice were immunized with NP-Ficoll. Serum and splenocytes were obtained after 7 days. A) Representative examples of gating for B220hi and B220lo. B,C) Frequencies of follicular (FO) and B-1 cells in spleen. D,E) Levels of NP-specific IgM and IgG in serum. F,G) Frequency of NP-specific IgM and IgG PCs in spleen. H,I) Frequency of IgG3+ B220hi (B-2) and IgG3+ B220lo (B-1) B cells in spleen. Data are shown as median with each symbol representing an individual mouse (n=5 per group). Asterisks indicate significant differences (*p<0.05; **p<0.01; ***p<0.001) obtained using Two-way ANOVA with Bonferroni posthoc test.

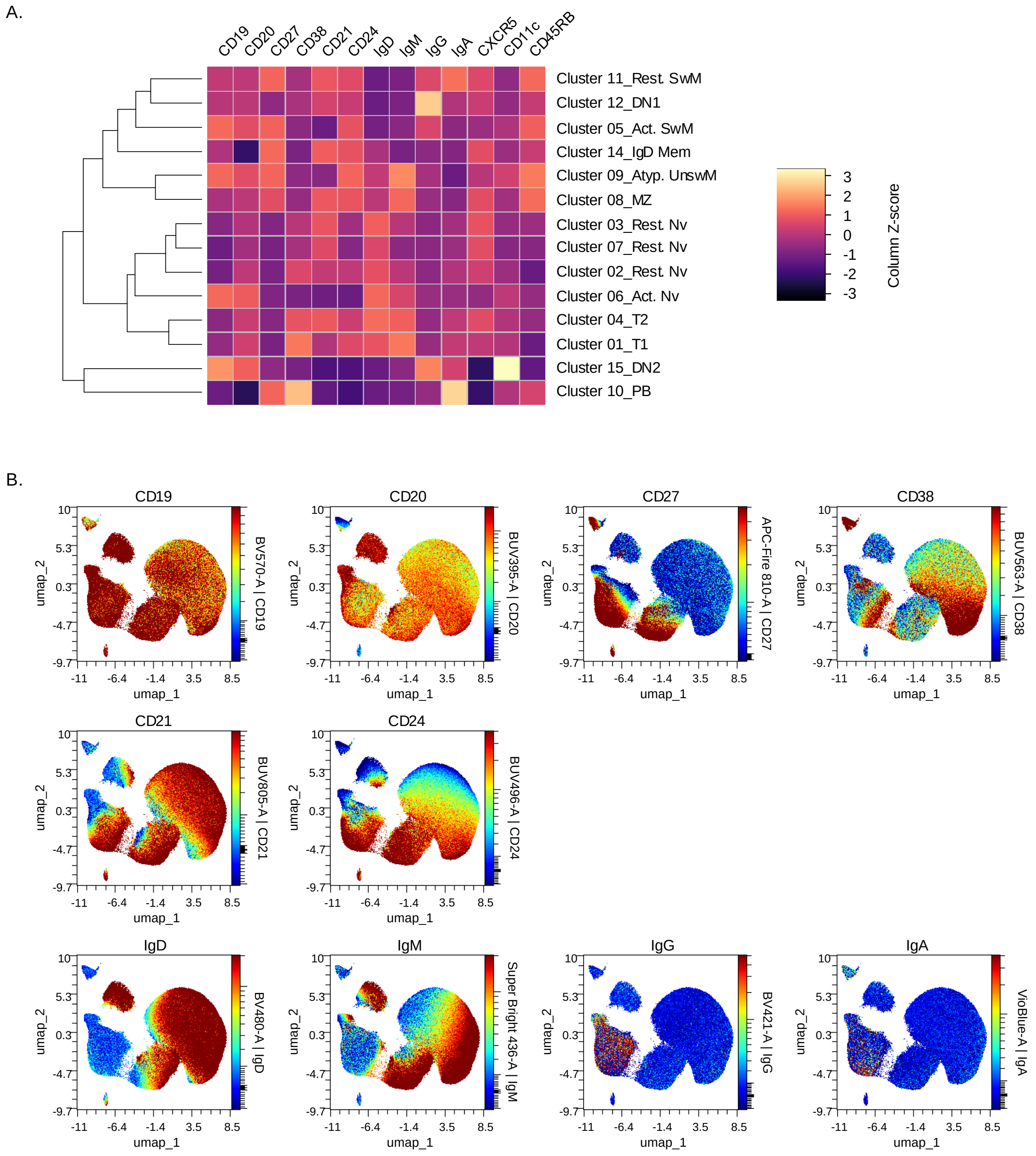

Figure S10. Expression of key markers in UMAP plots.

High dimensional spectral flow cytometry and FlowSOM clustering was used to identify multiple B cell subsets within PBMCs from SLE patients (n=15) and healthy donors (n=10). A) C) Heatmap showing expression levels of markers (columns) for each FlowSOM cluster (rows). Z-score was calculated using the median expression among all samples. B) UMAP was performed on gated live B cells using CD19, CD20, CD21, CD24, CD27, CD38, IgD, and IgM as

input parameters (neighbors = 15, mindist = 0.2). UMAP plots here show concatenated data from all samples (downsampled to 500k events).

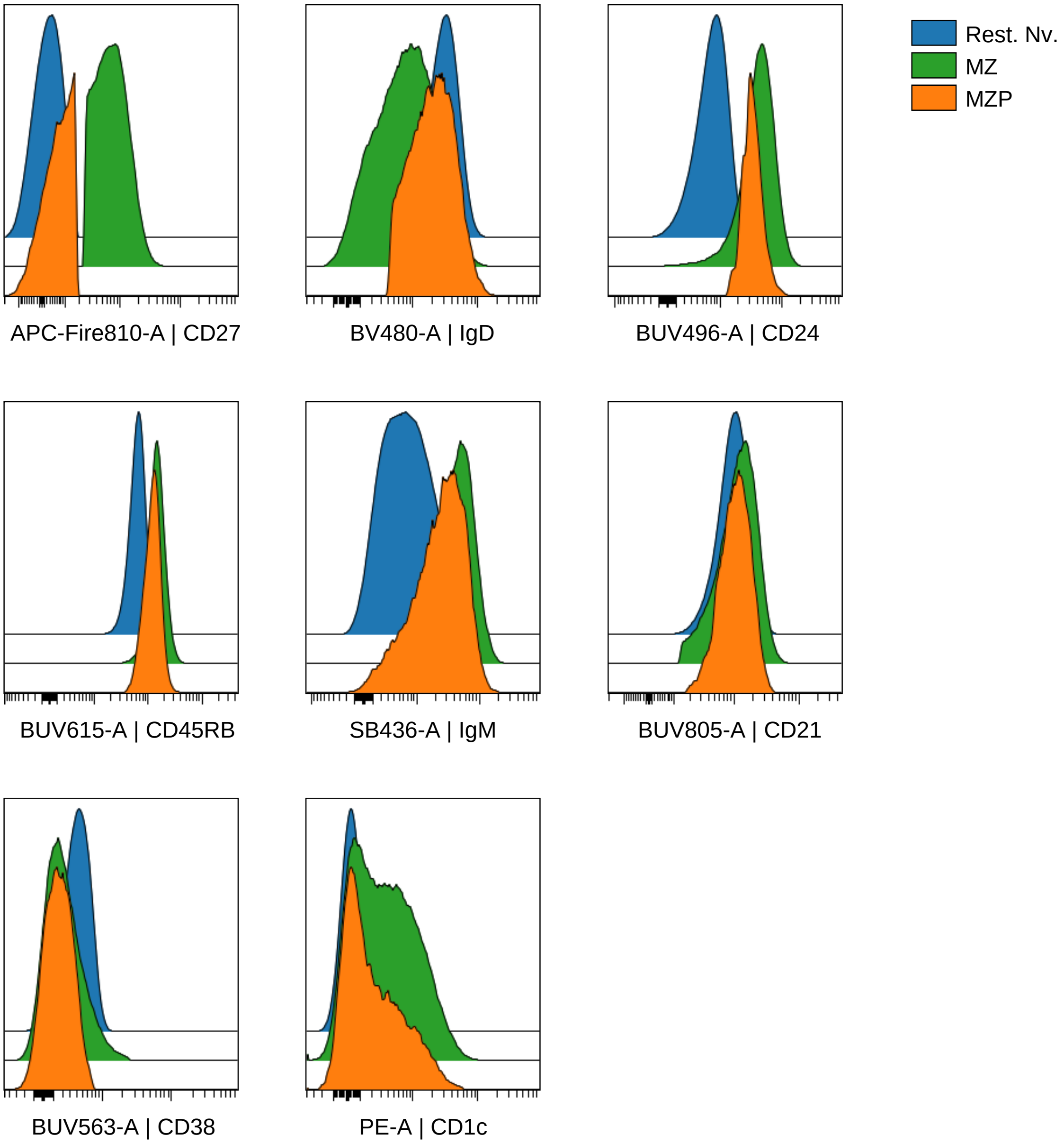

Figure S11. Phenotype of MZP.

High dimensional spectral flow cytometry was used to identify multiple B cell subsets within PBMCs from SLE patients (n=15) and healthy donors (n=10). Resting Naïve B cells (Rest. Nv.) were gated as Live CD19+ CD27- IgD+ CD38lo CD21+CD24lo cells. MZ B cells (MZ) were gated as Live CD19+ CD27+, IgMhi, CD21+ B cells. MZ precursors (MZP) were gated as Live CD19+ CD27- IgD+ CD38lo CD21+CD24hi cells. Histogram overlay plots here show concatenated data from all samples.

UMAP was performed using CD19, CD20, CD21, CD24, CD27, CD38, IgD, and IgM as input parameters (neighbors = 15, mindist = 0.2). UMAP plots here show concatenated data from all samples (downsampled to 500k events).

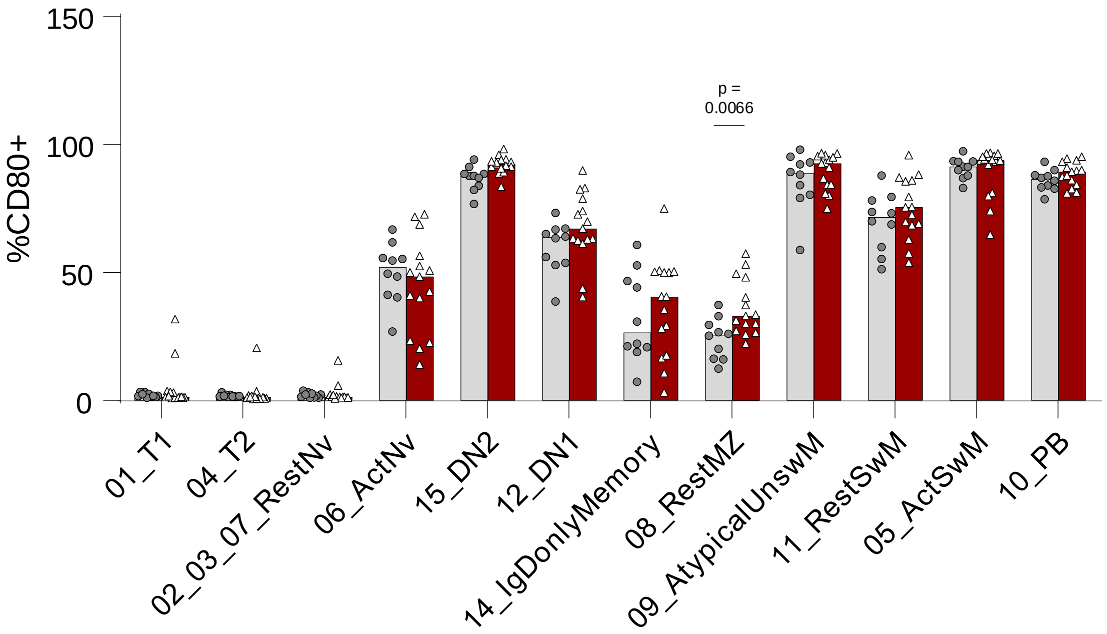

Figure S12. CD80 expression in all B cell subsets.

High dimensional spectral flow cytometry was used to identify multiple B cell subsets within PBMCs from SLE patients (n=15) and healthy donors (n=10). B cell subsets were identified using FlowSOM clustering as shown in Figure 6B. Expression of CD80 was analysed using Mann Whitney U test. Only significant p value is shown.

Table S1: Flow cytometry antibodies

Target species	Antigen	Fluorochrome	Clone	Company	Catalog Number
Mouse	B220	PE-eF610	RA3-6B2	eBioscience	61-0452-82
Mouse	CD5	BV421	53-7.3	Biolegend	100629
Mouse	CD11c	AF700	N418	Biolegend	117319
Mouse	CD19	BV711	6D5	Biolegend	115555
Mouse	CD21	PerCp-Cy5.5	7E9	Biolegend	123416
Mouse	CD23	BV786	B3B4	BD Biosciences	563988
Mouse	CD23	FITC	B3B4	Biolegend	101605
Mouse	CD23	PECy7	B3B4	eBioscience	25-0232-82
Mouse	CD24	PE-Cy7	M1/69	eBioscience	25-0242-80
Mouse	CD32b	APC	AT130-2	eBioscience	17-0321-82
Mouse	CD32b	PE	AT130-2	eBioscience	12-0321-80
Mouse	CD38	BV711	90/CD38	BD Biosciences	740697
Mouse	CD38	PE	90/CD38	BD Biosciences	553764
Mouse	CD43	FITC	S7	BD Biosciences	561856
Mouse	CD80	FITC	16-10A1	BD Biosciences	561954
Mouse	CD86	AF700	GL-1	Biolegend	105024
Mouse	CD93	BV421	AA4.1	BD Biosciences	747716
Mouse	CD93	PE	AA4.1	eBioscience	12-5892-81
Mouse	CD93	PE-Cy7	AA4.1	BioLegend	136506
Mouse	CD95	BV421	Jo2	BD Biosciences	562633
Mouse	CD95	PE-Cy7	Jo2	BD Biosciences	557653
Mouse	CD138	BV786	281-2	BD Biosciences	740880
Mouse	GL7	eF450	GL7	eBioscience	48-5902-82
Mouse	GL7	FITC	GL7	Biolegend	144604
Mouse	IAd	APC	AMS-32.1	eBioscience	17-5323-80
Mouse	IgA	FITC	C10-3	BD Biosciences	559354
Mouse	IgD	APC-Fire750	11-26c.2a	Biolegend	405744
Mouse	IgD	PE	11-26C.1	BD Biosciences	558597
Mouse	IgG1	BV605	A85-1	BD Biosciences	563285

Target species	Antigen	Fluorochrome	Clone	Company	Catalog Number
Mouse	IgG1	FITC	A85-1	BD Biosciences	553443
Mouse	IgG2	BV711	R2-40	BD Biosciences	744296
Mouse	IgG2	BV786	R2-40	BD Biosciences	744297
Mouse	IgG3	BV421	R40-82	BD Biosciences	565808
Mouse	IgG3	BV605	R40-82	BD Biosciences	744135
Mouse	IgM	APC-eF780	II/41	eBioscience	47-5790-82
Mouse	IgM	eF450	II/41	eBioscience	48-5790-82
Mouse	IgM	PE	II/41	eBioscience	12-5790-81
Mouse	IgM	PE-Cy7	eB121-15F9	eBioscience	25-5890-82
Mouse/ Human	Erk1/2 (pT202/pY204)	AF647	20A	BD Biosciences	612593
Mouse/ Human	Syk (pY348)	PE	I120-722	BD Biosciences	558529
Human	CD3	BV510	UCHT1	Biolegend	300447
Human	CD14	BV510	M5E2	Biolegend	301841
Human	CD19	PE-CF594	HIB19	BD Biosciences	562321
Human	CD19	PerCp-eF710	SJ25C1	eBioscience	46-0198-42
Human	CD27	BV421	M-T271	BD Biosciences	562513
Human	CD32	PE	FLI8.26	BD Biosciences	550586
Human	CD38	BV711	HIT2	Biolegend	303528
Human	CD80	BB515	L307.4	BD Biosciences	565008
Human	CD86	PE	2331 /FUN-1	BD Biosciences	555658
Human	HLA-DR	BV605	L243	Biolegend	307640
Human	IgA	PE-Vio770	IS11-8E10	Miltenyi	130-114-003
Human	IgD	AF700	IA6-2	Biolegend	348229
Human	IgD	PE-CF549	IA6-2	BD Biosciences	562540
Human	IgG	BV605	G18-145	BD Biosciences	563246
Human	IgG	BV785	G18-145	BD Biosciences	564230
Human	IgM	FITC	MHM-88	Biolegend	314506
	Mouse IgG1,k isotype control	AF647	MOPC-21	Biolegend	400130

Target species	Antigen	Fluorochrome	Clone	Company	Catalog Number
	Mouse IgG1,k isotype control	BB515	X40	BD Biosciences	564416
	Mouse IgG1,k isotype control	PE	(P3.6.2.8.1	eBioscience	12-4714-82
	Mouse IgG2a,k isotype control	APC	eBM2a	eBioscience	17-4724-81
	Mouse IgG2a,k isotype control	BV605	G155-178	BD Biosciences	562778

Table S2: Spectral flow cytometry antibodies

Target species	Antigen	Fluorochrome	Clone	Company	Catalog Number
Human	CD3	eFluor 506	UCHT1	eBioscience	69-0038-42
Human	CD14	eFluor 506	61D3	eBioscience	69-0149-42
Human	CD19	BV570	HIB19	Biolegend	302236
Human	CD27	APC-Fire810	QA17A18	Biolegend	393214
Human	CD38	BUV563	HB7	BD	741446
Human	CD21	BUV805	B-ly4	BD	742008
Human	CD24	BUV496	ML5	BD	741143
Human	CD20	BUV395	2H7	BD	563782
Human	IgD	BV480	IA6-2	BD	566138
Human	IgM	SuperBright436	SA-DA4	eBioscience	62-9998-42
Human	IgG	BV421	G18-145	BD	562581
Human	IgA	VioBlue	IS11-8E10	Miltenyi	130-113-479
Human	CD45RB	BUV615	MT4 (6B6)	BD	751482
Human	CD138	BV711	MI15	Biolegend	356521
Human	CXCR5	BV750	J252D4	Biolegend	356941
Human	CD11c	FITC	3.9	Biolegend	301604
Human	HLA-DR	PE-Fire810	L243	Biolegend	307683
Human	CD86	BV650	IT2.2	Biolegend	305427

Target species	Antigen	Fluorochrome	Clone	Company	Catalog Number
Human	CD40	PerCpCy5.5	5C3	Biolegend	334315
Human	CD80	APC-R700	L307.4	BD	565157
Human	CD69	PE-Fire640	FN50	Biolegend	310959
Human	CD1d	PE-Cy7	51.1	Biolegend	350309
Human	CD197	APC-Fire750	G043H7	Biolegend	353245
Human	CD1c (BDCA1)	PE	AD5-8E7	Miltenyi	120-000-889
Human	CD32B/C	AF647	4F5	Biolegend	Custom labeled by Biolegend

Table S3: Demographic and clinical characteristics

	SLE (n=15)	Healthy donor (n=10)	P value
Age	55 (21-76)	51 (28-66)	0.803[1]
Sex (n f/m (% female))	13/2 (87%)	9/1 (90%)	1.000[2]
Age at diagnosis	33 (13-54)		
Disease duration	24 (2-42)		
SLEDAI-2K	8 (0-24)		
Clinical symptoms			
Mucocutaneous	10 (67%)		
Arthritis	5 (33%)		
Renal	3 (20%)		
Other	4 (27%)		
Previous renal disease (n (%))	6 (40%)		
Class	Class III/IV, IV[3]		
Current medications			
PDN	4 (27%)		
HCQ	12 (80%)		
Immunosuppressants[4]	6 (40%)		

	SLE (n=15)	Healthy donor (n=10)	P value
Anti-dsDNA IgG			
IU/mL	14.1 (9.8-292.1)		
% positive	4 (27%)		
Complement			
C3 (g/L)	1.072 (0.673-1.425)		
C4 (mg/L)	209 (86-459)		
% low C3/C4	4 (27%)		
Platelets			
x10e3/uL	266 (86-391)		
% low (<100)	1 (7%)		
WBC			
x10e3/uL	6.28 (3.65-9.46)		
% low (<3)	0 (0%)		
Lymphocytes			
x10e3/uL	1.15 (0.49-2.81)		
% low (<1)	5 (38%)		

Table S3: Patient and healthy donor characteristics for samples included in spectral flow cytometry analysis. Values are shown as median with range in brackets, unless indicated otherwise.

[1]Obtained using Mann Whitney U test.

[2]Obtained using Fisher's exact test.

[3]One case of suspected lupus nephritis not biopsy-proven.

[4]These immunosuppressants include MMF, Azathioprine and Methotrexate.
